# Myasthenia gravis: from recognition of heterogeneity to a paradigm shift toward precision therapy

**DOI:** 10.3389/fimmu.2026.1929054

**Published:** 2026-09-22

**Authors:** Zhao-qing Li, Ting-yue Deng, Wen-jun Qiao, Wei-feng Xie, Yan-peng Huang, Fan-yu Liu, Qi Qin, Jing-sheng Zhang, Le Guan, Qing-feng Wang

**Affiliations:** 1 Liaoning University of Traditional Chinese Medicine, Shenyang, China; 2 The Affiliated Hospital of Liaoning University of Traditional Chinese Medicine, Shenyang, China

**Keywords:** biologics, biomarkers, complement inhibitors, FcRn antagonists, myasthenia gravis, neuroimmune disease, precision medicine, refractory myasthenia gravis

## Abstract

Myasthenia gravis (MG) is a heterogeneous neuroimmune disorder in which pathogenic autoantibodies impair neuromuscular junction transmission and produce fluctuating skeletal muscle weakness. Although conventional therapy with acetylcholinesterase inhibitors, corticosteroids, and broad immunosuppressants remains effective for many patients, its limitations are increasingly evident, including delayed onset, cumulative toxicity, and inadequate control in refractory disease. These challenges have accelerated the transition from non-selective immunosuppression to biologic and other targeted therapies. Recent advances in MG have revealed that treatment response is strongly shaped by disease heterogeneity across molecular, cellular, and structural levels. Antibody-defined subtypes, including acetylcholine receptor (AChR)-positive, muscle-specific tyrosine kinase (MuSK)-positive, low-density lipoprotein receptor-related protein 4 (LRP4)-positive, and seronegative MG, differ in immune mechanism, complement dependence, and therapeutic vulnerability. On this basis, neonatal Fc receptor (FcRn) antagonists, complement inhibitors, and B-cell-directed therapies have emerged as major biologic classes that increasingly enable mechanism-informed treatment selection. At the same time, resistance remains an important clinical problem, arising from persistent autoreactive immune compartments, pathway-level escape, and irreversible neuromuscular junction damage that may sustain disability despite immunological control. In this review, we discuss how immunological heterogeneity provides the biological rationale for targeted therapy in MG and synthesize current evidence for established and emerging biologics, including FcRn antagonists, complement inhibitors, B-cell- and plasma-cell-directed strategies, and cell-based immunotherapies. We further examine the major barriers that now define the field, particularly long-term safety, infection risk, treatment resistance, incomplete biomarker frameworks, and the distinction between active immune refractoriness and fixed structural impairment. Finally, we outline how biomarker-guided stratification, dynamic monitoring, and integration of neuroprotective or regenerative approaches may help move MG management from empiric escalation toward safer and more precise individualized care.

## 1 Introduction

Myasthenia gravis (MG) is an acquired autoimmune disorder of neuromuscular junction (NMJ) transmission mediated by pathogenic autoantibodies. Its global incidence is estimated at 4.1–30 cases per million person-years, with a prevalence of approximately 150–200 cases per million (1). Current standard treatment remains centered on symptomatic acetylcholinesterase inhibition, corticosteroids, conventional steroid-sparing immunosuppressants, and thymectomy in selected patients (2). Although these strategies have improved outcomes substantially, they are constrained by major clinical shortcomings. Long-term corticosteroid exposure causes considerable metabolic and infectious complications, conventional immunosuppressants often act slowly, and a meaningful subset of patients remains inadequately controlled despite prolonged therapy (2, 3). As a result, the therapeutic challenge in MG is no longer simply whether immune suppression is possible, but how to achieve disease control more rapidly, more selectively, and with less cumulative harm.

This therapeutic problem is inseparable from the biological heterogeneity of MG. At the molecular level, MG encompasses several antibody-defined subtypes, most prominently anti-acetylcholine receptor (AChR)-positive, anti-muscle-specific tyrosine kinase (MuSK)-positive, and anti-low-density lipoprotein receptor-related protein 4 (LRP4)-positive disease, together with seronegative forms that remain incompletely resolved (1). These subtypes differ fundamentally in their immunopathogenic logic. AChR-positive MG is largely mediated by complement-fixing IgG1 and IgG3 antibodies that promote receptor internalization and complement-mediated injury to the postsynaptic membrane (4). By contrast, MuSK-positive MG is typically driven by non-complement-fixing IgG4 antibodies that disrupt agrin-LRP4-MuSK signaling and compromise AChR clustering and NMJ stability (5). Clinically, MG is also heterogeneous in distribution of weakness, age at onset, thymic pathology, disease severity, and response to therapy (1). Taken together, these differences make clear that MG should no longer be viewed as a single therapeutic entity and provide the conceptual foundation for biomarker-guided precision management (6).

Beyond antibody status alone, accumulating evidence suggests that heterogeneity in MG extends across multiple biological layers that may directly shape treatment response. At the cellular level, persistent autoreactive B cells, plasmablasts, long-lived plasma cells, and dysregulated T-cell compartments contribute to disease chronicity and relapse (7). High-dimensional immune profiling has further identified pathogenic T-helper cell signatures associated with thymic immune dysregulation and disease severity, strengthening the view that MG activity cannot be fully explained by serum antibody detection alone (8, 9). At the structural level, prolonged immune injury may lead to irreversible remodeling of the NMJ, including simplification of postsynaptic folds, loss of receptor density, and broader synaptic degeneration (6). This distinction is clinically important because symptoms that persist in long-standing disease may reflect not only active immune pathology but also fixed structural damage that limits functional recovery even after immunological improvement.

These layers of heterogeneity help explain why refractory MG remains a major unmet clinical problem. Under chronic treatment pressure, pathogenic immune responses may persist through several mechanisms, including survival of long-lived plasma cells in immune-privileged niches, re-emergence of autoreactive plasmablasts, and sustained complement-mediated tissue injury despite conventional immunosuppression (7, 8). In parallel, clinical non-response may also reflect a mismatch between therapeutic target and disease biology—for example, complement-dependent effector injury is especially relevant in AChR-positive MG, whereas MuSK-positive disease follows a distinct non-complement-mediated mechanism (4, 5). Such observations support a shift away from empiric escalation toward biologically informed intervention and highlight the need to distinguish persistent immune activity from apparent refractoriness caused by accumulated structural damage (6).

Against this background, the development of novel biologics has reshaped the treatment landscape of MG. Pivotal phase 3 evidence for FcRn antagonists and complement inhibitors is summarized in Section 4 and **Table 1**. Here we emphasize that these agents provide clinical proof of concept for mechanism-matched therapy while also raising questions about response heterogeneity, retreatment, and long-term safety. B-cell-directed treatment has also emerged as a particularly relevant strategy in selected subtypes. Rituximab, for example, appears especially valuable in MuSK-positive disease, in which relapse biology is closely linked to ongoing B-cell and plasmablast activity (23).

**Table 1:** Established and emerging targeted therapies in MG: mechanism, evidence maturity, and best-fit clinical setting.

Therapeutic class	Representative agents	Mechanistic target	Strongest MG evidence	Best-fit clinical setting	Key limitations	Evidence level and key references
FcRn antagonists	Efgartigimod, rozanolixizumab	Reduce pathogenic IgG by blocking FcRn-mediated recycling	Approved/phase 3 evidence in generalized MG; strongest in AChR-positive disease, with class support extending to MuSK-positive disease	Rapid symptom control; steroid-sparing; recurrent or refractory generalized MG	Repeated treatment cycles; non-selective IgG lowering; incomplete control of upstream autoreactive compartments	Approved/phase 3 (10–14)
Complement inhibitors	Eculizumab, ravulizumab, zilucoplan	Interrupt terminal complement-mediated postsynaptic injury	Strongest evidence in refractory AChR-positive generalized MG	Biologically selected AChR-positive disease, especially severe or refractory settings	Pathway-specific infectious risk; limited relevance in MuSK-positive disease; no direct removal of autoantibody source	Approved/phase 3 and extension data (15–21)
Anti-CD20 B-cell depletion	Rituximab, ofatumumab	Deplete CD20-positive B-cell compartments and reduce plasmablast regeneration	Best cumulative signal in MuSK-positive MG; randomized support strengthened by RINOMAX	MuSK-positive disease; selected refractory or early generalized MG	Does not eliminate long-lived plasma cells; evidence heterogeneity across subtypes	Established but uneven by subtype (22–27)
Anti-CD19/plasma-cell-directed strategies	Inebilizumab, bortezomib, BCMA-oriented approaches	Target broader B-lineage compartments or plasma-cell survival	Mechanistically persuasive but still early for direct MG implementation	Highly selected refractory disease with persistent autoantibody production	Limited direct MG evidence; translational and toxicity concerns	Early translational/preclinical-heavy (28–33)
CAR-T and deeper immune-reset platforms	Anti-CD19 CAR-T, anti-BCMA CAR-T	Profound depletion of pathogenic B-cell or plasma-cell compartments	Exploratory for MG; proof of concept mainly extrapolated from hematologic malignancies and selected autoimmune diseases	Future option only for carefully selected, highly refractory disease within specialized research settings	CRS, ICANS, cytopenia, cost, manufacturing complexity	Exploratory/frontier; not routine MG therapy (32, 34–39)
Neurorestorative and combination strategies	Rehabilitation, inspiratory training, agrin-LRP4-MuSK repair concepts	Address NMJ recovery, muscle preservation, and residual disability	Supportive clinical evidence for rehabilitation; regenerative and NMJ-repair approaches remain exploratory	Pseudo-resistance, residual weakness, incomplete functional recovery	Limited direct MG efficacy data for regenerative interventions	Supportive or exploratory (40–48)

AChR, acetylcholine receptor; BCMA, B-cell maturation antigen; CAR-T, chimeric antigen receptor T cell; FcRn, neonatal Fc receptor; MG, myasthenia gravis; MuSK, muscle-specific kinase.

Despite these advances, the biologic era has introduced a new set of challenges that now define the next phase of precision care in MG. Greater target specificity does not eliminate the need for careful clinical judgment; it rather changes the questions clinicians must ask. Which biologic class is best matched to a given immunopathogenic subtype? Which biomarkers are sufficiently robust to guide treatment selection, retreatment timing, and monitoring of response? How should long-term risks such as infection, immunoglobulin depletion, and pathway-specific toxicity be balanced against the benefits of early aggressive control? How should clinicians manage patients in whom immunological improvement does not translate into full functional recovery because irreversible NMJ damage has already developed? These issues place MG at the forefront of precision neuroimmunology and make it an especially informative model for the broader transition from broad-spectrum immunosuppression to targeted therapy in neuroimmune disease.

In this review, we examine how immunological heterogeneity and treatment resistance provide the biological rationale for targeted therapy in MG, and we synthesize current evidence for established and emerging biologic strategies. We focus on four interrelated themes: first, the mechanistic basis of MG heterogeneity across molecular, cellular, and structural levels; second, the clinical positioning of major biologic classes, including FcRn antagonists, complement inhibitors, and B-cell- or plasma-cell-directed therapies; third, the safety, biomarker, and resistance challenges that limit current implementation; and fourth, the future integration of cell-based immunotherapy, neuroprotective strategies, and biomarker-guided treatment algorithms. By framing MG through these intersecting dimensions, we aim to provide a more clinically actionable account of how targeted therapies can be deployed more precisely, more safely, and with greater long-term therapeutic coherence.

## 2 Immunological heterogeneity and precision stratification in myasthenia gravis

Myasthenia gravis (MG) is not a single clinical entity but rather a heterogeneous autoimmune syndrome mediated by multiple pathogenic autoantibodies. As highlighted in the “Introduction”, this heterogeneity is a major obstacle to precision therapy because patients who share a common clinical diagnosis may differ substantially in pathogenic mechanism, immunological drivers, and treatment responsiveness. From a pathobiological perspective, this diversity can be understood at three interrelated levels. At the molecular level, the autoantibody repertoire defines the principal disease subtypes. At the cellular level, abnormalities among immune-cell populations shape the upstream processes that sustain antibody production and relapse. At the structural level, cumulative injury at the neuromuscular junction (NMJ) reflects the downstream consequence of prolonged molecular and cellular dysregulation. These three levels are closely linked and together form the conceptual basis for biomarker-guided stratification in MG (1).

Importantly, these levels of heterogeneity should not be viewed as parallel observations of equal practical value. They rather represent a progression from relatively accessible subtype definition to deeper mechanistic interpretation and, ultimately, to the assessment of irreversible tissue consequences. For this reason, antibody-based classification currently remains the most clinically actionable starting point, whereas cellular and structural markers may provide further refinement in selected settings. The following sections consider each level, in turn, and then integrate them into a precision-stratification framework.

### 2.1 Molecular level: immunophenotypic classification based on the autoantibody repertoire

Among the different dimensions of heterogeneity in MG, the molecular level currently provides the most direct basis for clinical classification. Depending on target antigen specificity, MG can be divided into several molecular subtypes that differ in antibody subclass, pathogenic mechanism, and treatment responsiveness (1, 4). This classification is more than a descriptive taxonomy. It provides the first practical step toward precision stratification because the identity of the pathogenic antibody often predicts which immunopathogenic pathway is dominant and therefore which targeted therapy is most biologically rational.

#### 2.1.1 Anti-AChR antibody-positive MG

Anti-acetylcholine receptor (AChR) antibody-positive MG is the most common subtype, accounting for 80%–85% of generalized MG cases, with pathogenic antibodies predominantly belonging to the IgG1 and IgG3 subclasses (4). These antibodies damage the postsynaptic membrane through three principal mechanisms: complement-mediated lysis, cross-linking followed by antigenic modulation with receptor internalization and degradation, and direct blockade of the acetylcholine-binding site (1). Among these mechanisms, complement activation is of particular importance because it represents both a major driver of tissue injury and a therapeutically targetable pathway.

Recent evidence suggests that the complement-activating capacity of AChR autoantibodies is not uniform across patients. Some pathogenic clones exhibit substantially stronger complement-activating activity than others (49). This observation has important stratification implications. Although AChR positivity defines the subtype at a broad level, it may not fully capture the functional heterogeneity within that group. In other words, antibody positivity may serve as the entry criterion for classification, whereas the magnitude of complement engagement may help explain differences in disease severity, refractoriness, and response to complement inhibition. As detailed in Section 4, clinical evidence for terminal complement blockade supports complement-targeted treatment in biologically selected AChR-positive MG. Thus, in AChR-positive MG, molecular classification is not merely diagnostic, but directly informative for therapeutic selection.

#### 2.1.2 Anti-MuSK antibody-positive MG

Anti-muscle-specific tyrosine kinase (MuSK) antibody-positive MG accounts for a minority of generalized MG cases, but it represents a clearly distinct immunopathogenic subtype (4). In contrast to anti-AChR MG, pathogenic antibodies are predominantly of the IgG4 subclass and do not activate complement. They instead interfere with NMJ formation and maintenance by disrupting MuSK-LRP4 interaction and inhibiting agrin-induced MuSK phosphorylation (5). This difference in pathogenic mechanism helps explain why the clinical and therapeutic behavior of MuSK-positive MG often diverges from that of AChR-positive disease.

A notable feature of MuSK MG is that IgG4 pathogenicity depends on functional monovalency rather than classical bivalent antibody binding. Through *in vivo* Fab-arm exchange, bivalent IgG4 molecules become functionally monovalent, thereby preventing MuSK dimerization and downstream signaling (50). This non-canonical mechanism provides a coherent explanation for the poor response to cholinesterase inhibitors often observed in this subtype. The primary abnormality lies not in simple reduction of receptor number but in disruption of receptor clustering and synaptic organization. In addition, relapse biology in MuSK MG appears to differ from that in AChR-positive disease. Following rituximab-induced remission, re-emergent anti-MuSK IgG4 antibodies are thought to arise mainly from newly generated plasmablasts rather than from residual long-lived plasma cells (22). This distinction is clinically relevant because it suggests that repeated B-cell-directed intervention may be especially important in this subtype. Overall, MuSK-positive MG illustrates how antibody-defined classification can reveal not only a different mechanism of injury but also a distinct therapeutic logic.

#### 2.1.3 Anti-LRP4 antibody-positive and seronegative MG

Anti-LRP4 antibodies are typically identified in patients who are negative for both AChR and MuSK antibodies, although the reported detection rates vary across geographic regions and patient populations (4). Because LRP4 functions as the receptor for agrin and is essential for NMJ formation, anti-LRP4 autoimmunity has a plausible pathogenic basis. Clinically, this subtype tends to be associated with relatively mild manifestations and generally favorable responses to conventional immunotherapy (4). Compared with AChR- and MuSK-positive disease, however, anti-LRP4 MG remains less well characterized, and its boundaries are still partly shaped by methodological differences in detection.

This issue becomes even more apparent in seronegative MG (SN-MG). Approximately 5%–10% of generalized MG cases remain negative on standard testing and are therefore classified as seronegative (51). However, the increasing use of high-sensitivity techniques such as cell-based assays (CBAs) has shown that some of these patients harbor low-titer anti-AChR antibodies or antibodies against clustered AChRs that are not detected by routine methods (51). Thus, seronegativity is not always a stable biological category; in some cases, it reflects the current limits of assay sensitivity rather than a truly antibody-negative disease state. This observation has broader implications for precision stratification. It suggests that molecular classification in MG remains an evolving framework and that improvements in detection technology may continue to refine and, perhaps, reconfigure the current subtype map.

### 2.2 Cellular level: heterogeneity among immune cell populations

Although antibody-defined subtypes provide the most practical classification framework, they do not fully explain the diversity of clinical course and treatment response observed in MG. The molecular repertoire reflects the end product of a more complex cellular immune process. Accordingly, cellular heterogeneity provides a second layer of stratification, one that may help explain why similar antibody-defined subtypes can still differ in severity, persistence, and relapse tendency. In recent years, single-cell and high-dimensional immune profiling have begun to reveal that distinct B-cell and T-cell abnormalities underlie different forms of MG and may eventually provide biomarkers beyond antibody detection alone.

#### 2.2.1 B-cell subsets and ectopic germinal centers

B cells are central effector cells in MG and contribute to disease through clonal expansion, formation of ectopic germinal centers, and secretion of pathogenic autoantibodies (24). However, the dominant pattern of B-cell abnormality is not identical across MG subtypes. In anti-MuSK-positive disease, pathogenic antibodies are produced mainly by short-lived plasmablasts, a finding that is consistent with the tendency toward relapse after B-cell depletion and aligns with the mechanisms discussed in Section 2.1.2 (22). In contrast, in anti-AChR-positive MG, particularly in early-onset disease, B-cell pathology is more closely associated with ectopic germinal-center formation within the thymus.

The MG-associated thymus exhibits many features of a tertiary lymphoid organ, with enrichment of B cells, T follicular helper (Tfh) cells, and follicular dendritic-cell networks that support autoantigen presentation and anti-AChR antibody production (52). These observations suggest that B-cell pathology in MG is compartment-specific as well as subtype-specific. In practical terms, peripheral plasmablast activity appears to be more prominent in MuSK-positive MG, whereas thymic germinal-center pathology is a more characteristic feature of AChR-positive disease. This distinction may help explain the variable responsiveness of different MG subtypes to B-cell-directed therapies and further supports the view that “B-cell-driven disease” is not a uniform biological state.

#### 2.2.2 Three dimensions of T-cell dysregulation

Whereas B cells function as direct effectors, T cells play a predominantly regulatory and permissive role in the autoimmune cascade. In MG, T-cell dysregulation can be broadly understood in three dimensions: expansion of Tfh populations, skewing toward proinflammatory helper subsets, and impairment of regulatory T-cell (Treg) function. First, circulating CD4^+^CXCR5^+^PD-1^+^ Tfh-cell frequencies are significantly elevated in MG patients. Notably, this increase correlates with disease severity even more closely than serum anti-AChR antibody titers and tends to normalize after clinical improvement with immunotherapy (53). This finding supports the view that Tfh expansion is not simply an epiphenomenon, but may reflect active immune support for ongoing autoantibody production.

Second, high-dimensional single-cell mass cytometry has identified two MG-associated memory T-helper subsets, ThCD103 and ThGM, that are enriched in the thymus, reduced in peripheral blood, and rebound after thymectomy (9). This dynamic distribution suggests a close relationship between thymic immune microenvironment and systemic disease activity. Third, within the thymus of MG patients, CD4^+^CD25^+^ Treg cells are preserved in number but show markedly impaired suppressive function together with downregulation of Foxp3 (54). This observation is particularly important because it places regulatory dysfunction upstream of both helper-cell expansion and downstream B-cell activation. Taken together, these three dimensions indicate that T-cell heterogeneity in MG is not merely secondary to autoantibody production but may actively shape the persistence, amplification, and phenotype of the autoimmune response. At present, these cellular abnormalities are more informative mechanistically than they are operationally in daily clinical practice, but they likely represent an important direction for future biomarker development.

### 2.3 Structural level: irreversible alterations at the neuromuscular junction

If molecular and cellular heterogeneity reflect active disease biology, structural heterogeneity reflects its accumulated consequence. Although MG has traditionally been regarded as a functional disorder, this view becomes incomplete in patients with long disease duration, in whom prolonged immune attack may lead to irreversible structural injury at the NMJ (6). Under sustained complement-mediated injury and membrane attack complex (MAC) deposition, electron microscopy has demonstrated simplification of postsynaptic folds, reduction of AChR density, and reorganization of the postsynaptic cytoskeleton. In parallel, the NMJ, as a whole, may undergo degenerative remodeling, including presynaptic terminal retraction and fragmentation of postsynaptic AChR clusters (6).

These structural changes have clear clinical significance. They help explain why some patients continue to experience weakness despite falling antibody titers or apparent immunological control. In such cases, persistent symptoms may no longer reflect only active autoimmune injury but also fixed structural loss that is difficult to reverse. This layer of heterogeneity is therefore particularly relevant to treatment expectations and to the distinction between active refractory disease and residual disability. Unfortunately, structural biomarkers remain less developed than molecular or cellular ones. Although serum neurofilament light chain (sNfL) is elevated in MG compared with healthy controls, its correlation with clinical severity has been inconsistent, limiting its usefulness as a reliable individual-level monitoring tool (55). Structural biomarker discovery thus remains not only one of the weakest but also one of the most clinically necessary components of current precision stratification in MG.

### 2.4 Integrated strategy: biomarker-driven precision stratification

Ultimately, the three levels of heterogeneity discussed above must be integrated if precision therapy in MG is to move beyond broad conceptual appeal toward practical clinical application. No single biomarker can capture the full complexity of MG because each level reflects a different aspect of disease biology. Antibody-related biomarkers currently provide the most established foundation for stratification. Antibody type determines the broad therapeutic direction, whereas the clinical value of antibody titers varies by subtype: anti-MuSK titers correlate relatively well with disease activity, whereas anti-AChR titers show a weaker relationship with individual severity (4). Within AChR-positive MG, complement-activation biomarkers may offer additional refinement by identifying patients more likely to benefit from complement inhibition (56).

Cellular immune biomarkers provide a second level of information. Tfh-cell frequency, plasmablast proportions, and Treg function may help to characterize systemic immune status, estimate disease activity, and potentially guide the timing of repeat B-cell-depletion therapy (53, 54). Structural biomarkers, exemplified by sNfL, remain exploratory but point toward a future framework in which cumulative tissue injury can be assessed alongside active immune dysfunction (55). These categories are therefore complementary rather than competing: antibody markers define subtype, complement-related markers refine effector-pathway stratification, cellular markers reflect immune activity, and structural markers may help quantify irreversible damage.

At the present stage, however, these biomarkers do not have equal clinical maturity. Antibody-based stratification is already established in practice, whereas complement-related and cellular markers remain more selectively informative, and structural markers are still under development. This uneven maturity is important to acknowledge because it defines the current limits of precision medicine in MG. Even so, the overall direction is clear. As multidimensional data on antibody specificity, complement activity, cellular immune status, and structural injury become increasingly integrated, especially in combination with multi-omics approaches and machine-learning-based prediction models, a more precise framework for subtype recognition and treatment-response forecasting may emerge (6). For now, the most realistic approach is layered stratification, in which antibody-defined subtyping serves as the clinical foundation and additional biomarkers are incorporated progressively as evidence and standardization improve.

Taken together, the immunopathological landscape of MG can be understood as a continuum from molecular heterogeneity and cellular dysregulation to structural degeneration of the NMJ. **Figure 1** summarizes these subtype-specific mechanisms and illustrates how the balance between active immune dysfunction and cumulative structural injury may shape both therapeutic opportunities and the limits of reversibility.

**Figure 1:**
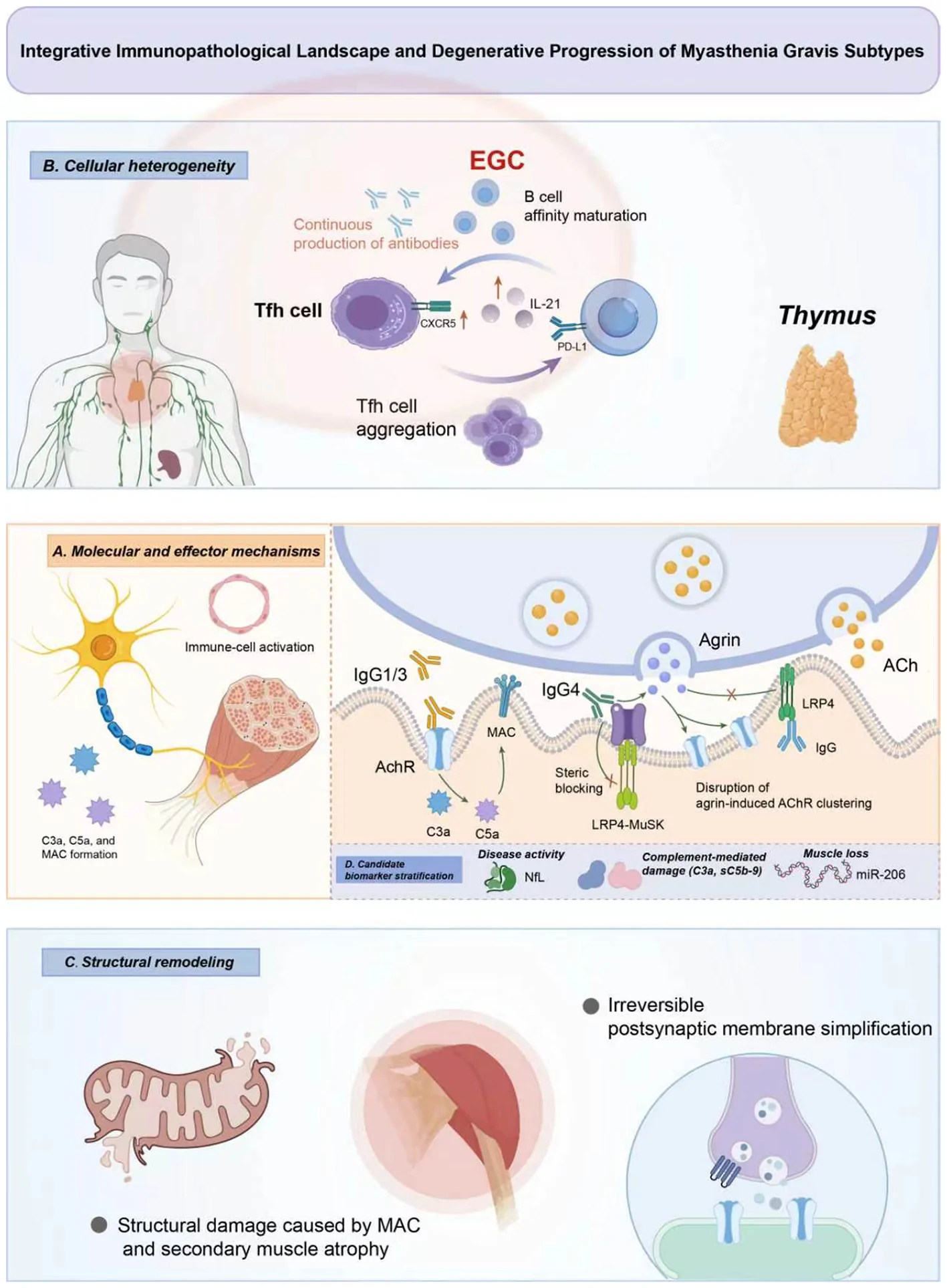
Integrative immunopathological landscape and degenerative progression of myasthenia gravis subtypes. **(A)** Central pathogenic mechanisms at the neuromuscular junction, including complement-mediated injury in AChR-antibody-positive disease and IgG4-mediated disruption of agrin-LRP4-MuSK signaling. **(B)** Cellular heterogeneity, emphasizing thymic ectopic germinal center activity, T follicular helper-cell aggregation, and sustained autoantibody production. **(C)** Degenerative remodeling, including irreversible postsynaptic membrane simplification and secondary muscle atrophy. **(D)** Biomarker-oriented stratification concept. Original illustration by the authors.

## 3 Mechanisms of therapeutic resistance in myasthenia gravis: immune escape, target-level bypass, and structural degeneration

Building on the heterogeneity framework outlined in Section 2, therapeutic resistance in myasthenia gravis (MG) can be understood as the clinical consequence of incomplete control across several pathogenic layers. In practice, the label “refractory MG” is often used to describe patients who remain symptomatic despite standard immunotherapy. However, this term can obscure substantial biological diversity. Some patients continue to generate pathogenic antibodies despite treatment, others exhibit incomplete response because the dominant effector pathway is not adequately blocked, and still others remain weak because structural damage at the neuromuscular junction (NMJ) has already become partially irreversible. Therapeutic resistance in MG should therefore not be viewed as a unitary phenomenon, but rather as a composite state in which immune persistence, target-level limitations, and cumulative tissue injury may coexist in varying proportions (6, 7).

This distinction is clinically important because the optimal response to non-response depends on the underlying mechanism. Escalating immunotherapy may be appropriate when active immune escape is evident, but the same strategy is less likely to be beneficial when symptoms are driven mainly by fixed synaptic or muscular damage. For this reason, a mechanistic view of resistance is essential to precision treatment. In the following sections, resistance in MG is discussed across three interrelated layers: immune escape, target-level bypass or pharmacological limitation, and “pseudo-resistance” caused by structural degeneration of the NMJ.

### 3.1 Immune escape

Immune escape refers to the persistence or reconstitution of pathogenic immune activity despite ongoing treatment. In MG, this phenomenon is especially important because most available therapies suppress parts of the autoimmune response rather than eliminate its full cellular basis. Long-lived plasma cells, newly generated plasmablasts, ectopic germinal centers, and memory lymphocyte populations may all sustain disease activity after apparently adequate immunosuppression. Thus, resistance at this level often reflects not failure of a drug in principle, but incomplete interruption of the cellular circuits that maintain autoantibody production (7).

#### 3.1.1 Long-lived plasma cells and newly generated plasmablasts

Long-lived plasma cells (LLPCs) are a sustained source of pathogenic antibodies in autoimmune disease. These cells can persist for months to years within immune-privileged niches such as the bone marrow, independent of ongoing antigenic stimulation or continuous replenishment from circulating B cells (28). Because LLPCs generally do not express, or express only very low levels of, CD20, they are intrinsically resistant to anti-CD20 therapies such as rituximab. This feature provides one of the clearest mechanistic explanations for why B-cell depletion is often effective but rarely curative with a single treatment course.

At the same time, the cellular basis of resistance is not identical across MG subtypes. In anti-muscle-specific kinase (MuSK)-positive MG, recurrent pathogenic antibodies after rituximab-induced remission appear to be produced mainly by newly generated plasmablasts rather than by bone-marrow LLPCs (22). This distinction is important because it suggests that relapse in MuSK-positive disease may reflect renewed B-cell differentiation rather than simple survival of a deeply entrenched plasma-cell reservoir. Clinically, this interpretation is consistent with the observation that rituximab can induce substantial benefit in MuSK-MG, yet relapses still occur in a subset of patients. In a multicenter, prospective, blinded review, 58% of anti-MuSK-positive patients achieved a Myasthenia Gravis Foundation of America Postintervention Status (MGFA-PIS) of minimal manifestations or better after rituximab (25). Nevertheless, the persistence of relapse indicates that initial depletion does not fully extinguish the pathogenic circuit. For this reason, cyclical rather than single-course B-cell depletion may be more effective in selected patients with MuSK-positive disease.

In AChR-positive or broader refractory MG, however, resistance is more likely to involve durable survival of plasma cells within supportive niches. Ex vivo reconstructions of the human bone-marrow microenvironment have shown that mesenchymal stromal-cell secretome, A proliferation-inducing ligand (APRIL), and hypoxic conditions can jointly maintain plasma-cell viability for prolonged periods (29). These survival signals may help explain why autoantibody production can persist despite otherwise effective immunosuppression. They also provide the rationale for targeting plasma-cell biology more directly. The proteasome inhibitor bortezomib has been shown in experimental autoimmune MG (EAMG) to deplete bone-marrow plasma cells, reduce anti-AChR antibody titers, and protect the postsynaptic membrane (30). Similarly, next-generation approaches directed against B-cell maturation antigen (BCMA), including CAR-T cells and bispecific antibodies, are being explored as ways to address plasma-cell-mediated resistance more directly. Collectively, these findings indicate that resistance to B-cell-directed therapy in MG should be interpreted in the context of which antibody-producing compartment remains dominant.

#### 3.1.2 Persistent activity of ectopic germinal centers

Ectopic germinal centers (EGCs) constitute a second major mechanism of immune escape. These tertiary lymphoid structures arise in chronically inflamed tissues and can serve as local sites of antigen-driven B-cell activation, somatic hypermutation, and immune-memory maintenance across several autoimmune diseases (57). In MG, EGCs are found predominantly in the thymus, especially in early-onset anti-AChR-positive patients (24). Such thymi display typical features of tertiary lymphoid organization, including proliferating B cells, follicular helper T (Tfh) cells, and networks of follicular dendritic cells (52). Importantly, B cells within thymic EGCs undergo somatic hypermutation, and the antibodies they generate are clonally related to circulating anti-AChR antibodies, strongly implicating the thymus as a central site of pathogenic antibody production (24).

This anatomical organization helps explain why thymectomy can be effective in selected MG subgroups. The MGTX phase III randomized trial showed that thymectomy plus prednisone was superior to prednisone alone over three years of follow-up (58). However, the benefit of thymectomy is not uniform across all patients. Late-onset MG is less frequently associated with thymic hyperplasia, and in this setting the contribution of the thymus to ongoing antibody production may be smaller. This suggests that the dominant reservoir of autoreactive immunity may shift from thymic to peripheral lymphoid compartments in different clinical contexts. Rituximab can partially disrupt EGC architecture through depletion of CD20-positive B cells, but it does not eliminate already differentiated plasma cells (24). Therefore, persistent EGC activity may continue to support immune escape even when circulating B cells are reduced. From a therapeutic standpoint, this mechanism highlights the importance of anatomical context in refractory disease and suggests that immune resistance cannot be explained solely by peripheral blood phenotypes.

#### 3.1.3 Persistence of immunological memory

A third layer of immune escape is the persistence of immunological memory. Even after clinical remission, patients with MG remain susceptible to relapse, a pattern that likely reflects the survival of long-lived memory B and T cells capable of rapid reactivation (7). Re-exposure to relevant antigens, intercurrent infection, or non-specific inflammatory triggers may be sufficient to rekindle pathogenic autoantibody production. This helps explain why the absence of overt symptoms does not necessarily imply that the autoimmune process has been extinguished.

In practical terms, persistence of immunological memory is one reason why many biologic interventions in MG function as disease-control strategies rather than definitive immune-reset therapies. It also helps explain why retreatment is often required even after an initially strong response. Viewed in this light, resistance is not always immediate treatment failure; it may also take the form of delayed reactivation after an apparently successful therapeutic interval. This concept is especially relevant in the era of biologics, where treatment schedules and maintenance strategies are increasingly tailored according to relapse kinetics rather than acute symptom burden alone.

### 3.2 Target-level resistance

Not all incomplete response in MG reflects persistence of the autoimmune source. In some patients, the dominant problem lies instead at the level of target engagement or pathway coverage. A biologic may successfully inhibit one key pathogenic node while leaving parallel or upstream disease-driving mechanisms relatively intact. This form of resistance is particularly relevant for highly specific targeted therapies, whose clinical strength, precision, is inseparable from a potential weakness, limited pathway breadth.

#### 3.2.1 Bypass activation of the complement cascade

The complement system is a major effector mechanism in anti-AChR-positive MG, and blockade of terminal complement activation has become an important therapeutic strategy. As summarized in Section 4 and **Table 1**, C5-directed therapy can meaningfully reduce disease burden in appropriately selected AChR-positive patients. Nevertheless, a subset of patients shows inadequate response or attenuation of benefit over time, raising the possibility of pathway-level bypass.

One explanation is that complement-mediated injury is not determined solely by C5 activation. The complement cascade comprises the classical, alternative, and lectin pathways, all of which converge at the level of C3 (59). When therapy blocks C5 downstream, upstream amplification loops may remain active and continue to generate pro-inflammatory fragments such as C3a, thereby sustaining inflammatory signaling beyond the direct reach of terminal pathway inhibition (59). This possibility has important implications for therapeutic refinement. It suggests that, in some patients, incomplete response to C5 blockade may not indicate absence of complement involvement but rather inadequate coverage of the most clinically relevant part of the cascade.

This rationale has driven interest in more proximal complement inhibitors. Pegcetacoplan, a PEGylated C3-targeting peptide, and C1s inhibitors act upstream of C5 and may, in principle, provide broader suppression of complement-mediated injury (60). At the same time, heterogeneity in the complement-activating capacity of autoantibodies may further influence who is most likely to benefit from this class of treatment (56). Thus, target-level resistance to complement inhibition should not be interpreted simply as drug failure. It may instead reflect mechanistic mismatch between the therapeutic node selected and the dominant complement dynamics in a given patient.

#### 3.2.2 Pharmacological limitations of FcRn antagonists

A related but distinct form of target-level limitation is seen with neonatal Fc receptor (FcRn) antagonists. By blocking IgG recycling, FcRn antagonists accelerate the degradation of pathogenic antibodies and have become an important therapeutic option in MG. As discussed in Section 4 and **Table 1**, this class has demonstrated clinical efficacy, but several intrinsic limitations may constrain the durability or extent of response.

First, FcRn antagonism is non-selective. It reduces all IgG subclasses, including protective antibodies, rather than selectively eliminating the pathogenic fraction (10). This broad mechanism contributes to its effectiveness, but it also introduces a pharmacological boundary and potential infection risk. Second, the effect is transient. IgG levels rebound after treatment cessation, and cyclical dosing is generally required to maintain response (11). In this sense, the limitation of FcRn antagonism lies not only in selectivity but also in temporality. The therapy lowers circulating pathogenic IgG efficiently, yet it does not directly eliminate the cellular sources that continue to produce it. Third, despite modest differences in FcRn affinity among IgG subclasses, robust individualized predictors of response are still lacking. At present, clinicians can identify patients who are likely candidates for FcRn blockade, but they are less able to predict who will show the most durable or complete response.

For these reasons, incomplete response to FcRn antagonists may reflect both biological and pharmacological boundaries. The strategy is powerful for rapidly lowering pathogenic IgG burden, but it does not by itself solve the deeper issue of ongoing autoantibody generation. Antigen-specific IgG degradation technologies, which aim to remove pathogenic antibodies while preserving the broader IgG pool, remain at an early stage of development. Their future progress may help distinguish transient disease control from more selective and sustainable immune modulation.

### 3.3 “Pseudo-resistance” arising from neuromuscular junction degeneration

The third layer of treatment resistance in MG is conceptually different from the first two. In immune escape and target-level bypass, the primary problem remains active autoimmune injury. In pseudo-resistance, by contrast, immunological activity may already be substantially controlled, yet symptoms persist because chronic injury has produced structural changes that are only partially reversible. This distinction is crucial because it shifts the clinical question from “why is the immune system still active?” to “how much recovery remains biologically possible?”.

#### 3.3.1 Irreversible structural damage

In chronic MG, sustained immune attack can result in irreversible structural alterations at the NMJ. Even when immunotherapy has reduced antibody levels or stabilized disease activity, persistent weakness may remain (6). This state should not be interpreted automatically as ongoing immune resistance. It rather may reflect cumulative tissue injury that has imposed a ceiling on functional recovery. Electron-microscopic and ultrastructural studies have shown that long-standing disease is associated with simplification of postsynaptic folds, reduced AChR density, and reorganization of the postsynaptic cytoskeleton; presynaptic nerve terminals may also undergo retraction and reduced branching (6, 61). Once established, these changes are not readily reversed by immunotherapy.

This concept has important therapeutic implications. It helps explain why some patients show discordance between immunological improvement and clinical recovery. It also introduces a boundary condition for biologic therapy: even highly effective immune-targeted treatment may have limited capacity to restore function once the synaptic substrate itself has been structurally compromised. In this context, therapeutic resistance is at least partly a problem of timing. Earlier intervention may prevent cumulative damage, whereas later escalation may control disease activity without fully restoring strength.

#### 3.3.2 Muscle atrophy and fibrosis

Structural degeneration in MG is not confined to the NMJ itself. Prolonged impairment of neuromuscular transmission can activate muscle-atrophy programs and lead to secondary tissue remodeling. Mechanistically, these processes involve ubiquitin-proteasome and autophagy-lysosome-mediated proteolysis, together with upregulation of atrophy-associated E3 ligases such as atrogin-1 and MuRF1 (62). Although the precise extent to which these pathways have been clinically mapped in MG remains less well defined than in other neuromuscular disorders, the general principle is biologically compelling: persistent transmission failure can eventually translate into muscle structural decline.

Evidence from broader neuromuscular disease cohorts reinforces this concern. Quantitative MRI studies have shown that increased intramuscular fat fraction is associated with worse functional outcome and that structural muscle alterations may progress even after disease activity is controlled (63). By analogy, chronic MG may follow a similar trajectory in selected patients. Repeated cycles of inflammation and incomplete repair may also promote fibrosis, which, in turn, could impair tissue penetration of subsequent therapies and further limit reversibility. Thus, pseudo-resistance in MG may arise not only from synaptic degeneration but also from downstream muscular remodeling that becomes increasingly independent of the original immune trigger.

#### 3.3.3 Clinical distinction between true and pseudo-resistance

Distinguishing immune-mediated true resistance from structure-driven pseudo-resistance is one of the most consequential tasks in the management of refractory MG. The two scenarios may produce similar symptoms, but they call for fundamentally different therapeutic responses. High antibody titers together with ongoing complement activation suggest persistent immune activity and may justify escalation of immunotherapy, including biologic agents or rational combination regimens. By contrast, persistent weakness in the setting of low antibody burden and limited evidence of ongoing immune injury points more strongly toward structural damage, in which case further intensification of immunotherapy is less likely to yield substantial functional gain (6).

A reliable distinction therefore requires multimodal assessment. Immunological markers, such as antibody titers and complement activation products, provide information about current disease activity. Electrophysiological studies, including repetitive nerve stimulation and single-fiber electromyography, assess transmission failure more directly. Quantitative muscle MRI may contribute additional insight into tissue status, with prolonged T2 relaxation suggesting edema or inflammation and increased fat fraction suggesting irreversible atrophy (63). No single modality is sufficient on its own. A multidimensional assessment is rather needed to determine whether the dominant problem is persistent immune attack, incomplete pathway control, or accumulated structural degeneration.

From a broader perspective, this distinction also refines the meaning of refractory MG itself. A patient may be “refractory” because the autoimmune system remains biologically active, because the chosen target incompletely captures the dominant effector pathway, or because long-standing disease has already left a structural legacy that limits recovery. Recognizing these possibilities is essential for individualized treatment planning. **Figure 2** summarizes these layered mechanisms of resistance and illustrates how true resistance and pseudo-resistance may overlap but remain mechanistically distinct.

**Figure 2:**
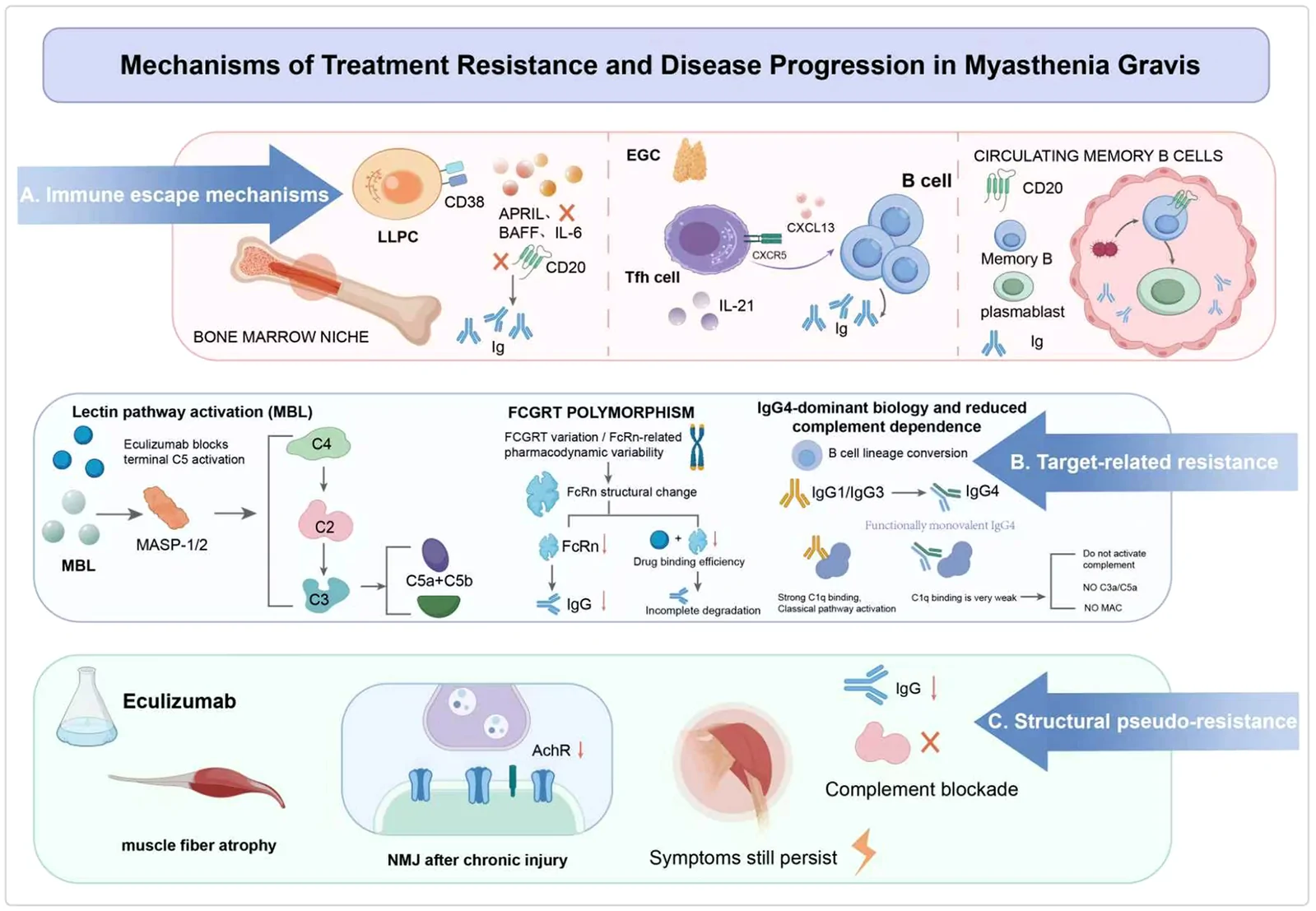
Mechanisms of treatment resistance and disease progression in myasthenia gravis. **(A)** Immune escape through long-lived plasma cells, ectopic germinal center activity, and memory B-cell or plasmablast re-expansion. **(B)** Target-related resistance, including bypass complement activation, FCGRT-related effects on FcRn-directed therapy, and shifts toward IgG4-dominant biology. **(C)** Structure-driven pseudo-resistance, in which chronic NMJ injury and muscle atrophy limit symptomatic recovery despite immunological control. Original illustration by the authors.

## 4 Targeted therapeutic landscape in MG: established and emerging biologics

At present, the most mature targeted classes in MG include FcRn antagonists, complement inhibitors, and B-cell-directed therapies. These agents have already altered expectations for refractory generalized MG, particularly in anti-acetylcholine receptor (AChR) antibody-positive disease and, in selected contexts, in anti-muscle-specific tyrosine kinase (MuSK) antibody-positive disease (10, 11, 15–17, 23). At the same time, the biologic era has introduced a new set of clinical questions. Long-term infection risk, retreatment timing, unequal efficacy across antibody-defined subtypes, incomplete coverage of long-lived plasma-cell biology, and the distinction between immune-controlled disease and structurally fixed disability have all become central to treatment selection. In parallel, more exploratory approaches, including plasma-cell-directed therapy, CAR-T-cell strategies, and neuroprotective or regenerative interventions, are beginning to extend the field beyond conventional immune suppression. For this reason, a useful review of targeted treatment in MG should not simply catalogue novel agents but should distinguish among therapies that are already clinically established, therapies that are mechanistically persuasive but still selectively positioned, and therapies that remain promising but early.

**Figure 3**; **Table 1** summarize the current therapeutic landscape, evidence maturity, and best-fit clinical settings discussed in this section.

**Figure 3:**
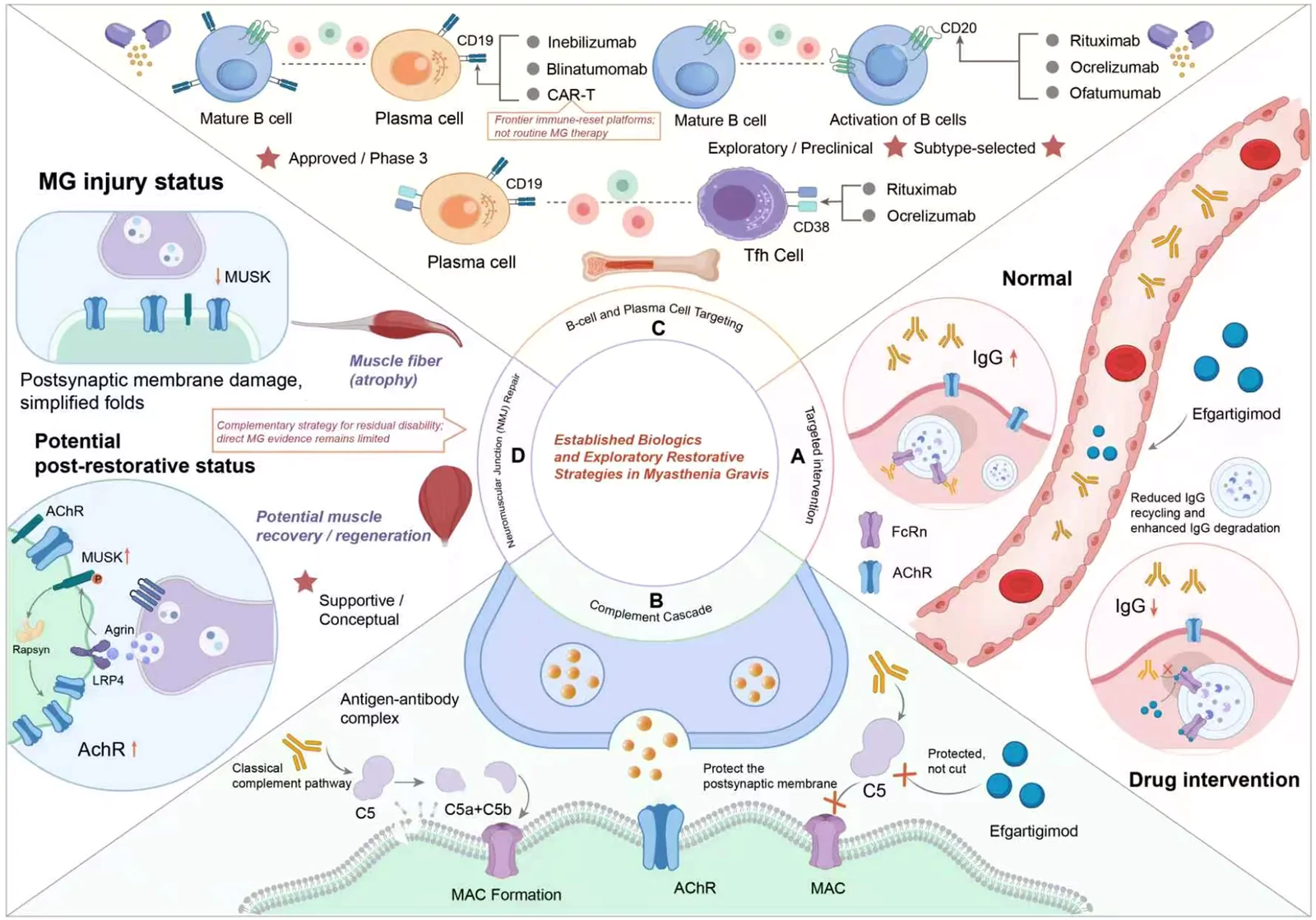
Established biologics and exploratory restorative strategies in myasthenia gravis. **(A)** Reduction of circulating pathogenic IgG through FcRn blockade. **(B)** Complement inhibition as a strategy to prevent membrane attack complex formation and protect the postsynaptic membrane. **(C)** B-cell- and plasma-cell-directed interventions, including CD20-, CD19-, and deeper immune-depletion approaches. **(D)** Neuromuscular junction repair, structural recovery, and muscle regeneration as a complementary restorative axis. The figure distinguishes approved or phase-3-supported biologic classes from exploratory immune-reset and neurorestorative strategies. Original illustration by the authors.

### 4.1 FcRn antagonists

#### 4.1.1 Biological rationale

Neonatal Fc receptor (FcRn) antagonists accelerate IgG degradation by blocking FcRn-mediated recycling. This mechanism gives FcRn antagonists several practical advantages. First, the effect on circulating autoantibody burden is rapid, making this class attractive when timely symptom improvement is needed. Second, FcRn antagonism acts upstream of subtype-specific effector injury in the sense that it lowers total IgG irrespective of whether pathology is mediated mainly by complement activation, antigenic modulation, or interference with neuromuscular junction (NMJ) signaling. Third, because FcRn blockade does not deplete B cells or plasma cells directly, it offers a strategy that is biologically distinct from B-cell depletion and can be integrated into broader treatment algorithms rather than viewed simply as another immunosuppressant. In a first-in-human study, efgartigimod reduced serum IgG by approximately 50% after single administration and by around 75% after repeated dosing, with recovery toward baseline over the following weeks and without measurable effects on serum albumin or non-IgG immunoglobulin classes (12). These pharmacodynamic properties established FcRn as a clinically tractable target and laid the mechanistic basis for later phase 2 and phase 3 programs.

Nevertheless, FcRn antagonism also has inherent biological limits. It reduces total IgG rather than selectively removing the pathogenic fraction, and it does not eliminate the cellular source of autoantibody production (12, 13). As a result, clinical benefit depends on repeated treatment cycles, and incomplete response may reflect ongoing production of pathogenic antibodies by plasmablasts or long-lived plasma cells even when circulating IgG is temporarily lowered. Thus, FcRn-targeted therapy is best understood as an efficient means of controlling pathogenic IgG burden rather than a strategy for immune reset.

#### 4.1.2 Efgartigimod

From a precision-therapy perspective, several points are worth emphasizing. First, the strongest evidence for efgartigimod currently lies in AChR antibody-positive generalized MG because that was the principal population in the pivotal program (11). Second, the therapeutic logic of FcRn blockade is broader than complement inhibition. Because it lowers pathogenic IgG regardless of complement dependence, it is conceptually relevant not only in AChR-positive MG but also in subtypes in which circulating autoantibody burden remains pathogenic through other mechanisms. Third, efgartigimod is especially valuable in the current MG landscape because it offers a rapid, targeted option without the cumulative toxicities typically associated with long-term corticosteroid escalation.

#### 4.1.3 Rozanolixizumab and class limitations

The subcutaneous route is also clinically relevant. In a chronic disease such as MG, treatment burden matters, and route-of-administration differences may influence long-term acceptability, health-system logistics, and patient preference. However, practical convenience should not obscure the same mechanistic limitations that apply to the class more broadly. FcRn antagonists reduce total IgG rather than selectively eliminating disease-specific antibodies, and their effect is intrinsically transient because antibody-producing cells remain in place (12, 13). For this reason, treatment with FcRn antagonists should be framed as cyclical biological control rather than curative intervention.

### 4.2 Complement inhibitors

#### 4.2.1 Pathogenic role of complement in MG

This pathogenic logic also helps explain why complement inhibition is not a universal MG strategy but a subtype-sensitive one. Complement-dependent tissue damage is central to many AChR-positive patients, yet it is far less relevant in MuSK-positive disease, which is typically mediated by IgG4 antibodies that do not activate complement (5). The value of complement inhibition in MG therefore lies not only in the availability of effective drugs but in the fact that it embodies the principle that antibody subtype should influence biologic selection.

#### 4.2.2 Clinical application of eculizumab and ravulizumab

Ravulizumab, a second-generation C5 inhibitor with a substantially prolonged half-life, extended this therapeutic concept while improving dosing convenience (16). In the phase 3 CHAMPION-MG study, ravulizumab significantly improved Myasthenia Gravis Activities of Daily Living (MG-ADL) compared with placebo and showed rapid onset with sustained effect across the treatment period (16). Long-term extension data further supported durability and safety (18). From a practical standpoint, the longer dosing interval is not a trivial issue. Treatment burden is a major determinant of long-term adherence and quality of life in chronic disease, and prolonged-interval dosing may enhance acceptability for patients requiring durable complement blockade.

Even so, complement inhibition has clear therapeutic boundaries. Its strongest rationale is in AChR-positive disease with active complement-mediated injury. It is not biologically expected to perform equally across all MG subtypes, nor does it eliminate upstream autoantibody production. Thus, complement inhibitors should be viewed as highly effective effector-pathway blockers in a defined mechanistic setting rather than as broad-spectrum biologics for all forms of MG.

#### 4.2.3 Patient selection and precision application

The broader implication is that complement inhibitors are among the most impressive examples of precision therapy already realized in MG, but their precision remains subtype-informed rather than fully biomarker-individualized. Future work will need to determine whether complement-related biomarkers can move selection beyond simple AChR positivity toward a more functionally stratified approach.

### 4.3 B-cell- and plasma-cell-directed therapy

#### 4.3.1 Anti-CD20 monoclonal antibodies

B-cell-directed therapy occupies a particularly important position in MG because B cells contribute not only to antibody production but also to antigen presentation, T-cell interaction, and maintenance of autoreactive immune memory (24, 52). Rituximab, the earliest anti-CD20 monoclonal antibody used in MG, remains the best studied in this category. Although much of the evidence has historically been observational, the cumulative signal has been strongest in MuSK-positive disease, where relapse biology appears closely linked to plasmablast generation from CD20-expressing precursor compartments (22, 25, 26). In a nationwide Austrian retrospective study including 56 rituximab-treated patients, complete stable remission was achieved more often in MuSK-positive than in AChR-positive disease, reinforcing the view that anti-CD20 therapy is especially valuable in MuSK MG (26). More recently, the RINOMAX randomized trial also strengthened the role of rituximab in generalized MG, showing meaningful efficacy in new-onset disease (23).

These findings are mechanistically coherent. In MuSK-positive MG, where pathogenic IgG4 production depends heavily on ongoing B-cell and plasmablast activity, depletion of CD20-positive compartments has a direct biological rationale. In AChR-positive disease, by contrast, anti-CD20 therapy may still be beneficial, but its effects are more likely to be limited by the survival of CD20-negative long-lived plasma cells. This distinction is central to proper clinical positioning. Rituximab should not be described simply as a generally effective biologic for all MG; its strongest and most consistent rationale lies in biologically selected subgroups, especially MuSK-positive disease.

Newer anti-CD20 agents such as ofatumumab are of theoretical interest because of alternative administration routes and pharmacological properties, but their direct evidence base in MG remains limited (64). Accordingly, these agents should currently be discussed as potential extensions of the anti-CD20 strategy rather than established MG therapies. For a *Frontiers*-targeted review, it is important to preserve this evidence hierarchy clearly.

#### 4.3.2 Anti-CD19 monoclonal antibodies and plasma-cell-directed therapy

One of the major limitations of anti-CD20 therapy is that it does not eliminate long-lived plasma cells, which often lack CD20 expression and can continue secreting pathogenic antibodies despite apparently adequate B-cell depletion (28, 29). This problem becomes especially relevant in refractory disease and in patients whose non-response appears to reflect persistent autoantibody production rather than only immune memory or incomplete target matching. CD19 is expressed earlier and more broadly across B-cell differentiation and persists on plasmablasts, making anti-CD19 therapy conceptually attractive as a deeper B-lineage intervention. Inebilizumab has already demonstrated efficacy in neuromyelitis optica spectrum disorder (31), and its biology makes it a plausible candidate for future MG application. However, direct MG evidence remains immature, so its discussion should remain prospective rather than practice-defining.

An even more direct route to overcoming plasma-cell-mediated resistance is plasma-cell targeting itself. Because long-lived plasma cells are central to persistent antibody production in some forms of refractory MG, agents that disrupt plasma-cell survival may address a pathogenic reservoir that conventional B-cell depletion leaves behind. The proteasome inhibitor bortezomib is the best-known example. In experimental autoimmune MG, bortezomib depleted plasma cells and reduced autoantibody levels (30). This is mechanistically important, but the current evidence is still largely preclinical or indirect. Therefore, plasma-cell-targeted therapy should be presented as a strong mechanistic strategy with limited direct clinical implementation in MG at present.

This distinction matters because the temptation in review writing is to make the logic appear more mature than the evidence. The field does not yet support routine plasma-cell-directed treatment in MG. What it does support is the conclusion that refractory disease cannot always be explained, or solved, at the level of conventional B-cell depletion alone.

#### 4.3.3 Exploration of CAR-T-cell therapy

However, translation from conceptual appeal to MG application remains at an early stage. Chimeric antigen receptor T-cell (CAR-T) therapy is not simply a more powerful version of rituximab. It introduces a different therapeutic scale, with the possibility of deeper immune reset and also major logistical, financial, and safety challenges. Cytokine release syndrome, immune effector cell-associated neurotoxicity, prolonged cytopenia, and infrastructure requirements all create a barrier that is far higher than for currently approved MG biologics. For this reason, CAR-T-based strategies should be framed as frontier or future-directed therapies rather than as near-term targeted options for routine refractory MG management. Their value in the current review lies in showing where the field may move next if tolerance-restoring or compartment-eradicating strategies become safer and more controllable.

### 4.4 Neuroprotective, restorative, and combination strategies

#### 4.4.1 Neuromuscular junction degeneration and therapeutic need

One of the most important boundary conditions in modern MG therapy is that immunological control does not always translate into full clinical recovery. As emphasized in Section 3, patients with long-standing disease may accumulate partially irreversible NMJ damage and secondary muscular remodeling (6, 61, 63). In such cases, biologics may suppress ongoing immune injury without restoring the structural substrate needed for normal transmission. This creates a therapeutic gap: as immune-targeted therapy improves, the residual burden of fixed synaptic or muscular damage becomes more clinically visible. Neuroprotective and regenerative strategies therefore deserve attention not because they replace immunotherapy but because they may eventually complement it in patients whose weakness reflects more than active autoimmunity.

#### 4.4.2 Neurotrophic factors and signaling pathway modulation

The agrin–LRP4–MuSK signaling axis is central to NMJ formation, maintenance, and repair (40). Because anti-MuSK and anti-LRP4 antibodies disrupt this pathway, strategies that stabilize or augment synaptic signaling are biologically attractive. Agrin-related approaches, modulation of downstream MuSK signaling, and interventions aimed at preserving postsynaptic organization all fit within this conceptual space, although direct therapeutic development remains limited. Similarly, myostatin inhibition and activation of hypertrophic signaling pathways such as Akt/mTOR are relevant to the broader goal of preserving or restoring muscle mass and function (41, 42). These approaches are not yet established MG therapies, but they address a dimension of disease that pure immunotherapy cannot fully resolve once structural change has accumulated.

#### 4.4.3 Rehabilitation and exercise intervention

Among the non-immunological strategies currently closest to clinical applicability, structured rehabilitation and exercise intervention already have practical value. Moderate aerobic and resistance training in stable MG has been shown to improve muscle performance without worsening disease activity, and inspiratory muscle training may be especially useful in patients with respiratory involvement (43, 44). These findings are important because they counter the older assumption that exertion should be broadly minimized in MG. Appropriately supervised rehabilitation instead appears capable of preserving function and helping patients translate immunological stabilization into practical physical benefit. In a precision-treatment framework, such interventions should not be treated as peripheral supportive care alone but as integral components of management for patients with residual weakness, deconditioning, or incomplete functional recovery.

#### 4.4.4 Combination treatment strategy

Taken together, the current biologic era in MG supports a layered treatment model rather than a single-class solution. International consensus guidance already recommends individualized, stepwise therapy based on disease severity, antibody status, and patient-specific context (2). In that framework, acute disease exacerbation may require rapid antibody lowering or effector blockade, using FcRn antagonists, complement inhibitors, plasma exchange, or intravenous immunoglobulin according to clinical urgency and disease biology. Longer-term disease control may then rely on B-cell-directed therapy, carefully selected conventional immunosuppression, or repeated biologic treatment cycles. For patients with accumulated structural damage, rehabilitation and, in the future, neuroprotective or regenerative interventions become increasingly relevant.

The key point is that combination does not simply mean using more therapies. It means aligning therapeutic mechanism with the dominant biological problem at a given stage of disease. In one patient, the main issue may be ongoing complement-mediated injury; in another, recurrent B-cell-driven relapse; and in another, persistent disability despite relative immune control because synaptic damage is already established. Thus, the future of MG therapy is likely to depend not only on the emergence of new agents but also on better clinical judgment regarding sequence, combination, retreatment timing, and the balance between immune suppression and functional restoration. To avoid repeating the safety narrative developed in Section 5, the emphasis here should remain on therapeutic sequencing, biological fit, and integration with structural-restorative strategies.

## 5 Safety, toxicity, and clinical risk management of targeted therapies in MG

The emergence of targeted therapies has altered the therapeutic logic of myasthenia gravis (MG), but it has not eliminated the problem of treatment-related risk. Rather than producing a simple shift from “toxic” to “safe” therapy, biologics redistribute risk into more class-specific patterns that must be anticipated and managed deliberately. This distinction is clinically important. Conventional corticosteroid-heavy regimens carry substantial cumulative metabolic, musculoskeletal, and infectious toxicity, whereas targeted agents more often introduce pathway-specific infectious vulnerability, immunoglobulin depletion, long-horizon immune effects, infusion or administration burden, and unresolved uncertainty regarding long-term implementation (2, 10, 11, 14–17, 19, 20, 23). Accordingly, safety in MG should not be discussed as an afterthought to efficacy but as a core dimension of precision treatment itself. The practical question is no longer simply which biologic can improve symptoms, but which biologic offers the most acceptable efficacy-safety balance for a given patient, disease stage, and therapeutic objective.

### 5.1 Reframing the benefit–risk balance in the biologic era

The central safety principle in modern MG management is that greater target specificity does not mean absence of risk. Each therapeutic class instead produces a distinct risk profile that must be interpreted in relation to disease subtype, severity, prior treatment exposure, infection susceptibility, and the expected duration of treatment. This is especially relevant in refractory generalized MG, where the clinical pressure to escalate therapy is often high. In such patients, the benefit of rapid disease control, steroid sparing, and rescue-therapy reduction may be substantial, but these gains must be balanced against class-specific hazards and the possibility that persistent weakness reflects structural pseudo-resistance rather than ongoing immune activity. In that setting, safety and biological appropriateness become inseparable: an unnecessary escalation is not merely ineffective but may also expose the patient to avoidable immunological and infectious risk.

For this reason, targeted therapy should be framed as safer in some respects than broad long-term immunosuppression and also more dependent on structured risk management. Complement inhibitors, FcRn antagonists, B-cell-directed therapies, and cell-based immune interventions differ not only in mechanism and efficacy but also in how they reshape host defense, retreatment strategy, surveillance burden, and tolerability over time. A clinically credible precision framework must therefore integrate efficacy and safety rather than treating them as separate decision layers.

### 5.2 Class-specific safety patterns and their clinical implications

#### 5.2.1 FcRn antagonists

FcRn antagonists have become attractive because they reduce pathogenic IgG rapidly without directly depleting B cells or plasma cells. This profile makes them appealing when fast symptom improvement is needed and when clinicians wish to avoid the cumulative systemic toxicities associated with prolonged corticosteroid escalation. At the same time, the mechanism is biologically non-selective: FcRn blockade lowers total IgG rather than removing only the pathogenic fraction. As a result, its safety profile is shaped less by organ toxicity than by immunological exposure over repeated treatment cycles (10–13, 19).

In practical terms, the principal concern is not a single dramatic toxicity signal but the need for infection vigilance, especially during long-term or repeatedly cycled treatment. The risk profile remains more favorable than that of many conventional immunosuppressive strategies, but it should not be described as risk-free. Repeated lowering of total IgG may be clinically acceptable in many patients, yet it creates a rationale for monitoring infectious events, cumulative treatment burden, and retreatment timing. Thus, FcRn antagonists are best understood as efficient controllers of circulating pathogenic IgG burden, with safety management centered on surveillance, individualized cycling, and avoidance of unnecessary over-treatment.

#### 5.2.2 Complement inhibitors

Complement inhibition has the clearest pathway-specific safety liability among currently established biologics in MG. Because terminal complement blockade compromises host defense against *Neisseria meningitidis*, susceptibility to meningococcal infection becomes the defining safety issue of this class (15–17, 20). This risk is sufficiently characteristic that it should be incorporated into treatment selection from the outset rather than addressed only after a drug has already been chosen. In other words, complement inhibitors are not simply “effective drugs with a warning”; they are biologically rational therapies whose benefit depends on disciplined infection-risk mitigation.

Vaccination is a mandatory component of this strategy before treatment initiation whenever feasible, but vaccination does not eliminate risk completely. Residual vulnerability can persist despite appropriate immunization, which means that patient counseling, symptom awareness, and rapid evaluation of febrile or meningitic presentations remain essential throughout treatment. This point has important implications for precision therapy. Complement inhibitors are most compelling in anti-AChR antibody-positive generalized MG with strong evidence of complement-mediated injury, but they are least attractive when the mechanistic match is weak or when the practical burden of ongoing infection-risk management outweighs the expected benefit. Thus, safety considerations reinforce, rather than compete with, biologically selective prescribing.

#### 5.2.3 B-cell-, plasma-cell-, and cell-based therapies

B-cell-directed therapy introduces a different temporal pattern of safety consideration. Here the issue is less an immediately recognizable pathway-specific infection syndrome than longer-horizon effects on humoral immune competence, retreatment timing, and the cumulative consequences of repeated immune depletion. Rituximab and related strategies are especially attractive in MuSK-positive disease and in selected refractory settings, but their implementation still requires attention to infection susceptibility, treatment sequencing, and the distinction between durable response and overextension of maintenance therapy (22, 23, 26, 27).

The safety horizon becomes even more important for therapies aimed at deeper immune resetting. Plasma-cell-directed strategies, anti-CD19 approaches, and CAR-T-cell therapy are conceptually attractive because they address cellular compartments implicated in persistent autoantibody production and treatment resistance. However, this increase in mechanistic depth is accompanied by greater translational risk. CAR-T-based approaches, in particular, should currently be framed as high-risk, high-complexity interventions rather than near-routine options for MG. Cytokine release syndrome, immune effector cell-associated neurotoxicity, logistical complexity, and cost all remain major barriers to broader use (32, 34, 35). For this reason, such therapies belong at the frontier of the field but not yet at the center of routine clinical implementation.

### 5.3 Pretreatment assessment and prevention

Risk management begins before the first dose is given. The pretreatment phase should establish whether the planned targeted therapy is biologically appropriate, clinically necessary, and practically supportable. At minimum, this assessment should integrate antibody-defined subtype, disease severity, prior immunotherapy exposure, history of recurrent infection, expected urgency of disease control, and the likelihood that current disability reflects active immune dysfunction rather than accumulated structural damage. This last distinction is particularly important in refractory MG. If symptoms largely reflect pseudo-resistance with fixed neuromuscular junction or muscle damage, the threshold for exposing a patient to additional immunological risk should be substantially higher.

For complement inhibitors, pretreatment assessment must also include vaccination planning and explicit patient education regarding meningococcal disease risk. For FcRn antagonists and B-cell-directed therapies, the emphasis is slightly different: the clinician must think prospectively about infection burden, retreatment cadence, and whether the intended benefit is symptom stabilization, steroid sparing, rescue reduction, or true re-design of long-term management. In all classes, pretreatment counseling should make clear that targeted therapy changes the pattern of risk; it does not abolish the need for monitoring.

### 5.4 On-treatment monitoring and adverse event management

Once biologic therapy is initiated, safety monitoring should run in parallel with efficacy assessment rather than after it. The practical aim is not merely to record adverse events but to determine whether the observed benefit remains proportionate to the biological and clinical burden of treatment. In MG, this balance may shift over time. A therapy that is justified during unstable refractory disease may become less clearly favorable once symptom control stabilizes, especially if repeated cycles, infectious events, or cumulative supportive care begin to dominate the treatment experience.

Monitoring should therefore be class-informed and clinically anchored. For FcRn antagonists, surveillance should focus on infection events, retreatment interval logic, and whether cyclical IgG lowering is still translating into meaningful functional benefit. For complement inhibitors, continued vigilance for meningococcal disease remains essential even after vaccination, and any suspicious infectious presentation should trigger immediate clinical evaluation. For B-cell-directed or deeper immune-depletion strategies, monitoring must consider both short-term tolerability and the longer-horizon consequences of repeated immune manipulation. In all cases, adverse-event management should be linked to treatment purpose: if a biologic is not producing durable functional gain, its risk tolerance should be reassessed rather than justified indefinitely by mechanism alone.

### 5.5 Long-term implementation, evidence gaps, and unresolved risk

The most important unresolved safety question in MG is not whether current biologics can be tolerated in the short term but how they should be implemented over years of disease management. Open-label extension studies and longer follow-up datasets have strengthened confidence in several established agents, but long-term use still raises questions regarding repeated exposure, treatment sequencing, class switching, cumulative infection burden, and patient selection outside tightly controlled trial populations (14, 18, 19). These uncertainties are especially relevant because targeted therapy in MG is increasingly moving from rescue-oriented use toward earlier and more individualized deployment.

This is also the point at which real-world evidence becomes particularly important. Clinical trials define short- to medium-term efficacy and broad tolerability, but long-term practice depends on how therapies perform across fluctuating disease states, mixed treatment histories, and imperfectly selected patients. Future work should therefore prioritize comparative effectiveness, long-term safety surveillance, retreatment optimization, and better identification of patients in whom efficacy gains justify sustained exposure. In that sense, safety is not merely a barrier to biologic use; it is one of the main dimensions through which the field will determine whether current targeted therapies truly deliver on the promise of precision medicine.

Taken together, targeted therapy in MG should be understood as a shift from generalized toxicity to differentiated risk. FcRn antagonists, complement inhibitors, B-cell-directed therapies, and emerging immune-reset strategies each offer important clinical opportunities, but each requires a different safety framework. The central challenge is therefore not simply to control disease more selectively, but to do so with risk-aware clinical judgment, structured monitoring, and realistic recognition of when further immunological escalation is unlikely to improve function. In modern MG care, efficacy, toxicity, and risk management are no longer separable topics; they are components of the same precision-therapy decision. **Table 2** summarizes the dominant safety concerns, pretreatment requirements, on-treatment monitoring anchors, and risk mitigation strategies across these therapeutic classes, discussed in detail below.

**Table 2:** Safety, toxicity, and clinical risk management of targeted therapies in MG.

Therapeutic class	Dominant safety concern	Pretreatment requirements	On-treatment monitoring	Risk mitigation approach	Key references
FcRn antagonists	Repeated lowering of total IgG with infection vigilance over cyclical treatment	Confirm biological fit, infection history, treatment objective, and need for repeated cycles	Track infectious events, treatment interval logic, and whether repeated IgG lowering still yields functional gain	Avoid over-cycling; align retreatment with symptoms and integrated monitoring rather than fixed escalation alone	(10–14)
Complement inhibitors	Meningococcal infection risk due to terminal complement blockade	Vaccination planning, patient counseling, and assessment of whether complement blockade is truly mechanistically justified	Urgent evaluation of febrile or meningitic symptoms; continued surveillance despite vaccination	Vaccination before initiation when feasible; structured education and rapid response planning	(15–20)
Anti-CD20 therapy	Longer-horizon effects on humoral immune competence and infection susceptibility	Review prior immunotherapy burden, infection history, and need for repeated depletion	Assess durability of benefit, timing of retreatment, and infectious complications during maintenance	Use subtype-appropriate positioning; avoid indefinite maintenance without clear functional benefit	(23–27)
Anti-CD19/plasma-cell-directed therapy	Deeper immune depletion with less mature safety characterization in MG	Reserve for strongly justified refractory settings; evaluate expected gain over better established options	Monitor closely for treatment-related toxicity and uncertain long-term tolerability	Treat as translational or trial-oriented when possible	(28–33)
CAR-T/immune-reset platforms	CRS, ICANS, cytopenia, logistics, and cost	Very careful patient selection, center capability, and risk-benefit review	High-intensity monitoring during and after infusion with neurotoxicity and systemic inflammatory surveillance	Not a routine MG option at present; should remain a frontier strategy	(32, 34–37)

CAR-T, chimeric antigen receptor T cell; CRS, cytokine release syndrome; FcRn, neonatal Fc receptor; ICANS, immune effector cell-associated neurotoxicity syndrome; MG, myasthenia gravis.

## 6 Precision implementation of targeted therapy in MG

If Sections 2–4 establish why myasthenia gravis (MG) is biologically heterogeneous and why targeted therapies differ in mechanism, then the next practical question is how those insights should alter treatment decisions at the bedside. Precision therapy in MG cannot be reduced to selecting the newest biologic. It rather requires a staged framework that integrates antibody-defined subtype, disease severity, tempo of progression, refractoriness risk, and the possibility that persistent weakness may reflect fixed neuromuscular junction (NMJ) damage rather than ongoing immune activity (1, 2, 6, 65). In this sense, precision treatment is not a static algorithm but a process of repeated biological reclassification.

In this review, precision therapy refers to an iterative treatment process in which therapeutic selection, monitoring, switching, and de-escalation are guided by the best available integration of antibody subtype, dominant immunopathogenic mechanism, disease severity, treatment tempo, safety risk, and evidence of reversible immune activity versus fixed structural damage. It does not imply that MG management is already fully biomarker-determined; it rather denotes a mechanism-informed framework that distinguishes clinically actionable evidence from exploratory or consensus-based decision support.

At present, the strongest clinically usable anchor remains antibody subtype. This determines the broad immunopathogenic logic of disease and therefore shapes which targeted class is most biologically plausible. However, subtype alone is not sufficient. Two patients with the same serological classification may differ in disease burden, relapse tendency, treatment urgency, and degree of structural irreversibility. For this reason, the most realistic current model is layered stratification. Antibody status provides the entry point, severity and refractoriness define escalation threshold, and dynamic monitoring determines whether treatment should be intensified, cycled, switched, or de-escalated. Importantly, these layers do not yet have equal evidence maturity. Antibody-based selection is already actionable in practice, whereas biomarker-guided retreatment, structural monitoring, and molecular prediction remain unevenly developed. A useful precision framework must therefore distinguish what is already clinically implementable from what is still conceptually attractive but not yet validated.

### 6.1 Building the treatment decision framework

#### 6.1.1 Antibody subtype-guided strategy

By contrast, MuSK antibody-positive MG follows a different pathogenic logic. Because MuSK antibodies are predominantly of the IgG4 subclass and do not activate complement, complement inhibitors are biologically less compelling in this subgroup (5). B-cell-directed therapy with rituximab instead appears particularly relevant, consistent with the central role of plasmablast and B-cell dynamics in MuSK-MG (22, 23, 26). FcRn antagonists may also be effective because they reduce circulating IgG regardless of complement dependence (10). Thus, subtype-guided precision care in MuSK-positive disease is not simply a matter of choosing a different drug but of recognizing that the dominant injury pathway is different from the start.

Patients classified as double-seronegative or antibody-negative on standard assays require a further level of diagnostic refinement. Cell-based assays substantially improve antibody detection in clinically suspected seronegative disease and can reclassify a proportion of patients who would otherwise remain in a biologically ambiguous category (51, 66). This point is more than technical. In a precision-treatment framework, an unresolved antibody status means an unresolved therapeutic logic. High-sensitivity testing therefore has practical value because it can move a patient from empiric management toward subtype-informed intervention. At the same time, it should be acknowledged that the seronegative category remains one of the least mature areas of current precision therapy. Some patients can be reclassified with better assays, but in others the underlying disease biology remains only partially resolved. Accordingly, treatment in this subgroup still relies more heavily on clinical phenotype and treatment response than in well-defined AChR- or MuSK-positive disease.

Overall, antibody subtype currently provides the most reliable first layer of treatment stratification. It does not predict everything, but it remains the clearest starting point for matching biologic class to dominant immunopathogenic mechanism.

#### 6.1.2 Stratification by disease severity

Ocular MG is generally approached first with symptomatic therapy and low-intensity immunomodulation, usually acetylcholinesterase inhibition with or without low-dose corticosteroids (2). However, more than half of ocular MG patients may generalize within two years, and whether very early immunotherapy reliably alters that transition remains incompletely resolved (67, 68). For that reason, ocular MG should not be treated as biologically trivial, but neither should it automatically trigger early biologic escalation in the absence of broader disease burden. The most defensible approach is careful monitoring with low threshold for reassessment when ocular disease is unstable, treatment-limiting, or suggestive of broader generalization risk.

In mild-to-moderate generalized MG, treatment still commonly begins with corticosteroids and conventional steroid-sparing agents, especially when symptom burden is manageable and rapid deterioration is not occurring (2, 69). Here targeted therapies may be introduced earlier in selected patients, but the decision is not yet fully standardized. The emerging logic is that biologics should be considered not only after prolonged failure but also when the anticipated toxicity of conventional escalation is high or when the underlying subtype strongly favors a biologic mechanism. Even so, the evidence base for very early biologic use is less mature than for their use in refractory disease.

Severe generalized weakness and myasthenic crisis represent a different treatment context altogether. In this setting, rapid control becomes the priority, and plasma exchange or intravenous immunoglobulin remains central because their effect is immediate relative to most background immunotherapies (70). Targeted biologics may then be integrated as part of medium-term stabilization, especially in biologically suitable refractory cases. This is particularly relevant in AChR-positive generalized MG, where complement inhibitors have the strongest evidence in refractory severe disease (15, 16, 19). Thus, severity stratification is not simply about symptom description; it determines whether the therapeutic goal is acute rescue, medium-term stabilization, or long-term disease redesign.

#### 6.1.3 Refractoriness risk and treatment escalation

A major challenge in modern MG management is determining when standard therapy is no longer sufficient and escalation should occur. Approximately 10%–20% of patients are considered refractory by conventional clinical definitions, although the biological meaning of refractoriness is often heterogeneous (6, 71). As outlined in Section 3, persistent symptoms may reflect ongoing immune escape, incomplete target coverage, or fixed structural damage rather than one uniform state. This distinction is essential because precision escalation should be based on mechanism, not only on treatment history.

In practical terms, patients with early severe disease, high treatment burden, repeated rescue therapy requirement, or poor tolerance of prolonged corticosteroid exposure should be recognized as higher-risk candidates for early escalation (71–75). However, escalation should not be equated automatically with simply “using stronger immunotherapy.” In AChR-positive refractory generalized MG, complement inhibition or FcRn antagonism may be the most rational next step, whereas in MuSK-positive disease rituximab may be more biologically coherent (5, 10, 11, 15–17, 23, 26). Conversely, in patients whose persistent weakness appears increasingly disconnected from active immune burden, repeated escalation may yield diminishing returns.

**Figure 4** is therefore best interpreted as a proposed precision framework rather than a fully validated treatment-sequencing algorithm. Its value lies in organizing current decision logic, while its limitation is that several branches remain supported more by convergent reasoning, consensus guidance, and mechanistic plausibility than by prospective comparative trials. **Table 3** complements this framework by consolidating these clinical contexts, dominant biology, reasonable targeted options, monitoring anchors, and key caveats into a single bedside implementation matrix.

**Figure 4:**
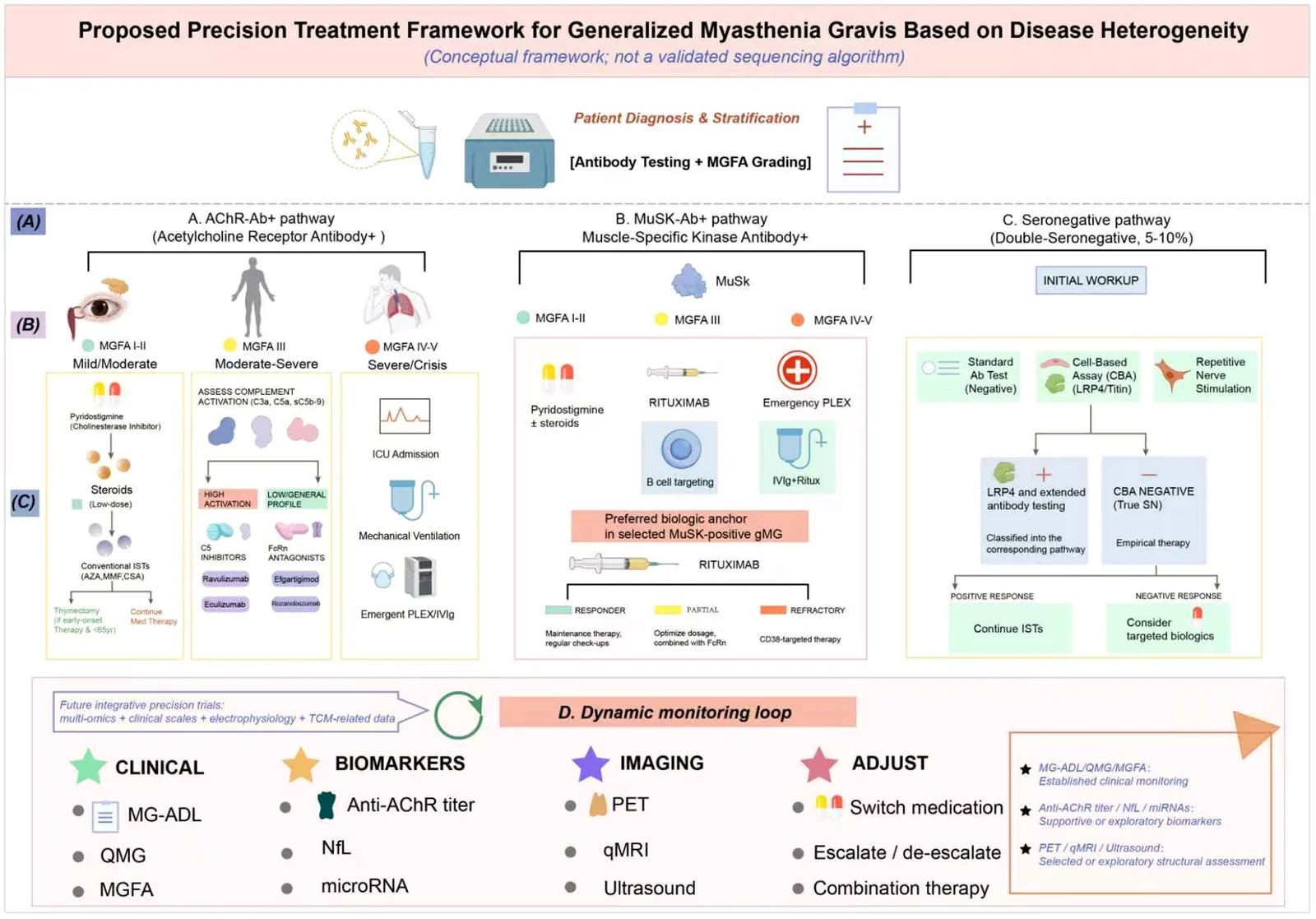
Proposed precision treatment framework for generalized myasthenia gravis based on disease heterogeneity. The figure begins with antibody testing and MGFA-based stratification and then separates **(A)** AChR-positive, MuSK-positive, and seronegative pathways, each further stratified by **(B)** MGFA-based severity state, with **(C)** corresponding pathway- and severity-specific treatment options and decision points. It concludes with **(D)** a dynamic monitoring loop integrating clinical scales, biomarkers, imaging, and treatment adjustment. This figure is intended as a conceptual implementation framework based on current evidence maturity, consensus guidance, and mechanistic plausibility, not as a fully validated bedside sequencing algorithm. Original illustration by the authors.

**Table 3:** Precision implementation matrix for targeted therapy in MG.

Clinical context	Dominant biology	Reasonable targeted options	Monitoring anchors	Key caveats	Key references
AChR-positive mild/moderate generalized MG	Complement-relevant autoantibody disease, but often still manageable with conventional therapy	Conventional immunotherapy first; consider FcRn antagonists earlier in selected patients with high steroid burden	MG-ADL, QMG, MGFA class, antibody status, treatment burden	Evidence for very early biologic use is less mature than for refractory disease	(2, 4, 10–14, 77)
AChR-positive refractory generalized MG	Persistent complement-mediated injury or incomplete control under conventional therapy	Complement inhibitors or FcRn antagonists; combination sequencing guided by phenotype and response	Clinical scales, rescue therapy need, selected complement-related markers, PK/PD-informed retreatment logic	Do not confuse ongoing activity with fixed structural disability	(10, 11, 14–21, 69, 80, 82, 87, 88)
MuSK-positive generalized MG	IgG4-dominant disease with strong B-cell/plasmablast relevance and little complement dependence	Rituximab is often the most coherent biologic anchor; FcRn antagonists may also be useful	Clinical status, relapse tendency, antibody-related data, need for repeat B-cell-directed therapy	Complement inhibitors are biologically less compelling in this subtype	(5, 10, 11, 22, 23, 25–27, 50, 81)
Seronegative or diagnostically evolving MG	Unresolved antibody biology and uncertain mechanistic fit	Refine diagnosis with cell-based assays, electrophysiology, and phenotype-led treatment	Clinical response, repeat serology/CBA, electrophysiology	Therapeutic logic remains less mature than in clearly defined AChR- or MuSK-positive disease	(2, 4, 6, 51, 66, 77, 79)
Severe generalized weakness or myasthenic crisis	Immediate functional threat with high urgency of disease control	PLEX/IVIg for rescue; targeted biologics as medium-term stabilization in suitable refractory biology	Respiratory status, ICU trajectory, MGFA severity, rescue-treatment response	Acute rescue logic differs from long-term biologic redesign	(2, 70, 72–74, 77, 88)
Persistent weakness with suspected pseudo-resistance	Residual structural damage, NMJ simplification, or secondary muscle remodeling; distinguish from active immune refractoriness	Rehabilitation, supportive management, and cautious avoidance of unnecessary immune escalation	Clinical scales plus electrophysiology, qMRI, and structural biomarkers where available	Escalation may add risk without restoring function if active immune injury is limited; requires integrated clinical and structural assessment	(6, 40–42, 45, 46, 61, 63, 65, 85, 86)

CBA, cell-based assay; ICU, intensive care unit; IVIg, intravenous immunoglobulin; MG, myasthenia gravis; MG-ADL, Myasthenia Gravis Activities of Daily Living; MGFA, Myasthenia Gravis Foundation of America; MGC, MG composite; PLEX, plasma exchange; PK/PD, pharmacokinetic/pharmacodynamic; qMRI, quantitative muscle MRI; QMG, quantitative myasthenia gravis.

### 6.2 Treatment monitoring and dynamic adjustment

#### 6.2.1 Multidimensional clinical assessment

Once targeted therapy has been initiated, precision treatment depends as much on monitoring as on initial selection. MG is dynamic, and treatment response often unfolds across different time scales. Symptoms may improve before structural recovery is possible, biomarkers may shift before measurable functional change, and residual weakness may persist despite immunological benefit (63, 76, 77). Accordingly, treatment monitoring must be multidimensional. No single clinical or laboratory parameter is sufficient to capture all therapeutically relevant changes.

In this context, monitoring serves three different functions. First, it determines whether disease activity is improving. Second, it helps distinguish pharmacological non-response from delayed but meaningful biological response. Third, it may reveal that the dominant problem has changed, for example from active autoimmune dysfunction to residual structural impairment. These distinctions become especially important in the biologic era, because treatment cycles, retreatment timing, and switching decisions often depend on fine-grained assessment rather than on crude relapse/no-relapse categories alone.

#### 6.2.2 Functional and quality-of-life scales

Standardized outcome scales remain the most established tools for longitudinal assessment in MG. MG-ADL is especially useful because it is patient-reported, clinically intuitive, and sensitive to change in daily functional burden (78). Quantitative MG (QMG) provides a more objective, examiner-based assessment of weakness, whereas the MG Composite (MGC) integrates physician-rated and patient-relevant elements into a broader clinical picture (79, 80). These tools are already deeply embedded in clinical trials and real-world follow-up and therefore remain the practical core of response monitoring.

Their importance in precision care is not diminished by the rise of biomarkers. They rather anchor interpretation. Biomarkers may indicate pathway modulation, but patients are treated for improved function, reduced crisis risk, and better quality of life. Thus, targeted treatment should not be judged solely by immunological change. A precision framework in MG still requires symptom scales and functional measures as the primary language of clinical benefit.

#### 6.2.3 Biomarker monitoring

Biomarker-guided treatment in MG is an area of genuine promise, but current maturity is uneven. Antibody-related information remains the most established layer. Antibody subtype is already clinically actionable, and in some settings antibody titer may add value, especially in MuSK-positive disease, where titers correlate more closely with disease activity than in AChR-positive MG (4, 81). Complement activation markers such as C3a, C5a, and soluble terminal complement complex are also biologically appealing, particularly for monitoring complement-directed therapy (21, 82, 83). However, their routine clinical use in MG remains limited, and most of the strongest support still comes from mechanistic or experimental settings rather than standardized patient-level implementation.

Cytokine profiles and broader immune signatures provide a second layer of information, potentially reflecting inflammatory burden or treatment effects (18), yet these markers are not currently mature enough to serve as stand-alone decision tools. Their clinical interpretation is complicated by biological variability, assay standardization issues, and uncertain threshold values. For this reason, they are better viewed at present as supportive or research-stage biomarkers rather than as definitive guides for routine treatment adjustment.

Markers of structural injury remain even more exploratory. Neurofilament light chain has attracted interest because of its broader relevance across neuromuscular disorders, but its precise role in MG monitoring remains unsettled (55, 84). This does not make such markers unimportant. On the contrary, biomarkers capable of distinguishing ongoing immune activity from cumulative structural damage would be among the most clinically valuable advances in the field. It simply means that this layer of precision monitoring remains under development.

#### 6.2.4 Imaging and electrophysiology

Imaging is less established in routine MG monitoring, but it may become increasingly relevant in the context of long-standing disease and pseudo-resistance. Quantitative muscle MRI can assess fat infiltration, edema, and muscle-volume changes and has already proved informative in other neuromuscular diseases (85, 86). In MG, its most plausible future role is not first-line diagnosis but structural phenotyping in patients whose symptoms and immune markers appear discordant. In other words, electrophysiology is currently closer to clinical implementation, whereas advanced imaging is more likely to become a complementary tool in selected refractory or structurally complex cases.

#### 6.2.5 Pharmacokinetic/pharmacodynamic-based dose optimization

For FcRn antagonists, total IgG decline and recovery provide an intuitive pharmacodynamic framework, and in principle pathogenic antibody reduction could further refine retreatment timing (12). However, the field should be cautious not to overstate current personalization. Although IgG kinetics clearly inform class behavior, robust individualized retreatment rules remain incompletely standardized. Similarly, complement inhibitors produce clinically meaningful improvement in many patients, but optimal long-term adjustment still depends more on integrated clinical response than on a fully validated biomarker-guided model (87, 88).

Thus, pharmacokinetic/pharmacodynamic-informed optimization is one of the most promising near-term directions in MG precision care, but it is still partly transitional. The concept is sound; the implementation is not yet fully mature. The most realistic current approach is to combine symptom scales, functional outcomes, and selected biological readouts rather than rely on any single pharmacodynamic marker.

#### 6.2.6 Liquid biopsy and molecular marker exploration

However, their current role should be described carefully. At present, most liquid-biopsy markers in MG remain exploratory. They are useful for hypothesis generation and may eventually contribute to relapse prediction, subtype refinement, or early treatment-response assessment, but they are not yet sufficiently standardized for routine treatment decisions (89–93). Multi-analyte platforms developed in oncology provide a methodological template rather than a directly transferable model (93). In MG, the most plausible future application would be a composite monitoring system in which antibody-related data, immune-cell signatures, circulating RNAs, and structural injury markers are interpreted together rather than in isolation.

Taken together, heterogeneity-based precision treatment in MG can be understood as a three-layered process. First, treatment direction is anchored by biologically meaningful stratification, especially antibody subtype, severity, and refractoriness profile. Second, efficacy is monitored through integrated clinical, electrophysiological, and biomarker-based assessment. Third, treatment is dynamically adjusted according to changing disease biology, not simply according to elapsed time or fixed escalation rules. This framework is already partly visible in clinical practice, but its full realization will require better biomarkers, stronger comparative evidence, and clearer distinction between active immune refractoriness and residual structural disability.

## 7 Therapeutic implications and future directions

The recent expansion of biologic and other targeted therapies has changed the treatment landscape of myasthenia gravis (MG) more profoundly than any development since the establishment of corticosteroids, steroid-sparing immunosuppressants, and thymectomy as core therapeutic strategies (1, 2). However, the significance of this shift lies not simply in the availability of more drugs. It rather reflects a broader change in therapeutic logic. MG is no longer being managed solely as a single autoimmune disorder requiring stepwise escalation of generalized immunosuppression. It is instead increasingly approached as a heterogeneous neuroimmune disease in which antibody subtype, effector mechanism, immune-cell persistence, and cumulative neuromuscular junction (NMJ) damage all influence treatment response and the limits of reversibility.

Viewed in this way, the biologic era in MG has produced two simultaneous advances. First, it has established that subtype-inwformed, mechanism-matched therapy can substantially improve outcomes in selected patient groups, especially in refractory generalized disease. Second, it has clarified that disease control and disease reversal are not synonymous. Even highly effective biologics may fail to restore function fully when structural injury has accumulated or when pathogenic immune compartments remain incompletely covered by the chosen target. For this reason, the current moment should not be described simply as therapeutic success. It is more accurately understood as a transition from broad immune suppression toward layered precision care, with not only new strengths but also new sources of complexity.

### 7.1 What current biologics have changed in clinical MG care

One of the clearest changes in modern MG management is the move away from purely empiric escalation. Conventional therapies remain indispensable and continue to benefit many patients, but they offer limited specificity, slow onset, and substantial cumulative toxicity when used long term (2, 77). Novel biologics have altered this picture by making it possible to intervene at defined pathogenic nodes. FcRn antagonists reduce circulating pathogenic IgG rapidly, complement inhibitors block a key terminal effector pathway in AChR-positive disease, and B-cell-directed therapies address upstream autoreactive cellular compartments, particularly in MuSK-positive MG (10, 11, 15–17, 23).

This does not mean that MG has already become a fully biomarker-driven disease. It rather means that the field now has several therapies whose use can be justified by recognizable biological logic. In AChR-positive generalized MG, complement-dependent injury provides a clear rationale for terminal complement inhibition (4, 15, 16). In MuSK-positive disease, the central role of B cells and plasmablast dynamics helps explain the strong positioning of rituximab and the relevance of FcRn antagonism (5, 10, 23, 26). Thus, one of the most important conceptual advances is that treatment selection is increasingly linked to subtype-specific mechanism rather than to symptom burden alone.

At the same time, the limits of this transition should be stated clearly. Current precision in MG is still layered and incomplete. Antibody subtype provides the strongest clinically usable entry point, but it does not fully resolve differences in relapse biology, durability of response, or likelihood of fixed structural impairment. Accordingly, biologics have not replaced clinical judgment; they have increased the need for biologically informed judgment.

The greatest practical impact of novel biologics has so far been seen in refractory generalized MG. This population historically carried the highest burden of prolonged steroid exposure, repeated rescue therapy, unstable disease control, and impaired quality of life (3, 6, 94, 95). Complement inhibitors and FcRn antagonists have now provided substantial new options for many of these patients, especially those with anti-AChR antibody-positive generalized disease (10, 11, 15, 16, 19). Similarly, rituximab has become one of the most important biologic strategies in MuSK-positive MG, where it often achieves durable benefit and in some patients may alter the long-term disease course more effectively than conventional steroid-sparing regimens (23, 25, 26).

An equally important point is that biologics have shifted the practical meaning of refractoriness. Historically, refractory disease often implied repeated failure of broadly suppressive therapy. In the current era, however, non-response must be interpreted more carefully. A patient may appear refractory because the dominant effector pathway is not being blocked, because autoreactive plasma-cell or memory compartments persist, or because long-standing NMJ damage has imposed a limit on recovery even when immune activity improves. Novel biologics therefore do more than provide rescue options; they force a more biologically specific interpretation of treatment failure.

### 7.2 Future research directions and therapeutic prospects

Several next-generation strategies are being explored to move beyond the current limits of FcRn antagonism, complement inhibition, and conventional B-cell depletion. Bispecific antibodies are of particular interest because they may provide more selective or multi-level targeting than existing monoclonal platforms (96–99). Potential strategies include selective depletion of autoreactive B-cell populations, more efficient coupling of cytotoxic effector cells to pathogenic B cells or plasma cells, and broader complement-pathway modulation that addresses bypass or incomplete pathway coverage. These concepts are scientifically attractive because they aim to improve precision without simply increasing generalized immunosuppression.

Small-molecule approaches are also of interest, especially when they target intracellular signaling pathways that are not readily addressed by conventional biologics (33, 100–102). BTK inhibitors, JAK inhibitors, next-generation proteasome inhibitors, and protein-degradation technologies such as PROTACs all offer theoretical relevance to MG because they may modulate B-cell activation, cytokine signaling, or plasma-cell survival. However, for MG, these approaches remain mostly exploratory. Their current value lies in expanding the therapeutic imagination of the field rather than in near-term clinical readiness. For a review aimed at *Frontiers in Immunology*, it is important to preserve that distinction clearly.

A more ambitious goal is the development of treatments capable of deeper immune reset or restoration of antigen-specific tolerance. In MG, anti-CD19 or anti-BCMA CAR-T approaches are especially relevant in principle because they address compartments implicated in persistent autoantibody production and treatment resistance (32, 34–37). However, their current evidence base in MG remains largely indirect and extrapolated from hematologic malignancies and selected autoimmune diseases. These strategies should therefore be framed as high-risk, high-concept platforms rather than near-term standard biologics.

Antigen-specific tolerance induction, gene editing, and engineered regulatory-cell approaches remain attractive because they point beyond chronic suppression toward deeper disease redesign (38, 39, 103–106). At present, however, their evidence base is still predominantly preclinical or indirect for MG, so they should be presented as plausible future directions rather than clinically ready solutions.

As emphasized in Sections 3–5, one of the major unresolved problems in MG is how to treat patients whose functional burden reflects accumulated structural damage rather than only active immunity. This makes neuroprotective and regenerative medicine an important future direction, but the evidence gap should be acknowledged explicitly. Interventions that stabilize the agrin–LRP4–MuSK axis, support NMJ repair, promote muscle regeneration, or reduce secondary muscular degeneration may eventually become critical partners to immune-targeted therapy, especially in long-standing disease, but most remain exploratory for MG at present.

At present, however, this area remains less mature than the biologic field. Neurotrophic modulation, stem-cell-based support strategies, and agents promoting muscle regeneration are all biologically plausible, but their direct clinical evidence in MG is limited (45–48). Their current value is therefore conceptual and strategic. They help define what targeted therapy in MG still cannot do and identify where future combination treatment may be needed. This is especially important because a biologic that controls immune activity may still leave a patient functionally impaired if no parallel strategy exists to address the structural consequences of past disease.

Artificial intelligence and data-driven prediction models are increasingly discussed in relation to precision medicine in neuroimmune disease (107–110). In MG, the most realistic value of such tools lies not in replacing clinician judgment, but in improving classification, monitoring, and treatment matching. Models integrating antibody subtype, clinical scales, electrophysiology, imaging, and biomarker profiles may eventually help identify patients at risk of crisis, treatment failure, or early relapse. Similarly, digital symptom tracking and wearable monitoring may make longitudinal fluctuation more visible in routine care.

However, this area should be presented carefully. AI does not yet solve the core biological uncertainties of MG, and current models remain dependent on the quality and standardization of their underlying clinical and molecular data. Therefore, artificial intelligence should be framed as an enabling layer for future precision implementation, not as a primary therapeutic solution in itself. Its importance will depend on whether it can improve real treatment decisions rather than merely produce more complex predictions.

Future precision-medicine trials in MG should also move beyond single-modality stratification. An integrative precision medicine design could combine antibody subtype, clinical scales, electrophysiology, imaging, immune-cell profiling, circulating molecular markers, and carefully standardized traditional Chinese medicine (TCM)-related treatment data. Such studies would be particularly valuable for identifying which patients may benefit from combining biologics with botanical or other adjunctive therapies, with the goal of reducing cumulative drug exposure, dosage burden, and treatment-related toxicity. Importantly, this strategy should be evaluated prospectively and mechanistically so that TCM-related variables contribute to patient stratification and response prediction rather than remaining descriptive background information (111, 112).

### 7.3 Concluding remarks

Taken together, the contemporary treatment era in MG can be understood as a transition from broad immune suppression toward biologically stratified disease control. FcRn antagonists, complement inhibitors, and B-cell-directed therapies have already demonstrated that treatment can be matched more closely to subtype-specific immune logic, especially in refractory generalized disease. This represents a genuine therapeutic advance and has materially improved the outlook for many patients. At the same time, these successes have also made the remaining challenges more visible. Long-term safety management, incomplete biomarker maturity, persistence of pathogenic immune reservoirs, unequal response across subtypes, and the burden of fixed NMJ and muscle damage all limit the extent to which current biologics can be considered fully precision-guided or fully disease-modifying.

The next phase of progress in MG is therefore likely to depend on three linked developments. First, current biologics must be integrated more intelligently through better subtype recognition, monitoring, and retreatment strategy. Second, future immune-targeted platforms, including plasma-cell-directed therapy, bispecific antibodies, and immune-reconstitution approaches, must be judged not only by efficacy but also by safety, scalability, and appropriateness for defined patient groups. Third, treatment of MG must increasingly account for the structural and functional consequences of long-standing disease, which means that neuroprotective, rehabilitative, and regenerative strategies will need to be incorporated into the therapeutic framework rather than discussed separately from it.

In this sense, the central message of the field is no longer simply that MG can be immunologically suppressed. It is that MG management is becoming not only progressively more selective, more biologically informed, and potentially more durable but also more dependent on accurate stratification and realistic interpretation of treatment limits. The most credible future for precision therapy in MG lies not in any single breakthrough but in the integration of targeted immunotherapy, structured monitoring, risk-aware safety management, and support for synaptic and muscular recovery.
